# Rapid mid-Cretaceous diversification of squid and cuttlefish preceded radiation into coastal niches

**DOI:** 10.1038/s41559-026-03009-1

**Published:** 2026-03-30

**Authors:** Gustavo Sanchez, Fernando Ángel Fernández-Álvarez, Ainhoa Bernal, Elizabeth Heath-Heckman, Raphael Lami, Margaret McFall-Ngai, Michele Nishiguchi, Spencer Nyholm, Oleg Simakov, A. Louise Allcock, Daniel S. Rokhsar

**Affiliations:** 1 Molecular Genetics Unit, Okinawa Institute of Science and Technology, Onna, Japan; 2 Centro Oceanográfico de Gijón (IEO, CSIC), Spanish Institute of Oceanography, Gijón, Spain; 3 Institut de Ciències del Mar (ICM-CSIC), Barcelona, Spain; 4 Centro Oceanográfico de Baleares (IEO, CSIC), Spanish Institute of Oceanography, Palma, Spain; 5 Departments of Microbiology, Genetics, and Immunology, and Integrative Biology, Michigan State University, East Lansing, MI USA; 6 Laboratoire de Biodiversité et Biotechnologies Microbiennes (LBBM), Observatoire Océanologique, CNRS, UPVD, UMR8176, Sorbonne Université, Banyuls-sur-Mer, France; 7 Department of Biosphere Sciences and Engineering, Carnegie Science, California Institute of Technology, Pasadena, CA USA; 8 Department of Molecular and Cell Biology, University of California, Merced, CA USA; 9 Department of Molecular and Cell Biology, University of Connecticut, Storrs, CT USA; 10 Department for Neurosciences and Developmental Biology, University of Vienna, Vienna, Austria; 11 School of Natural Sciences and Ryan Institute, University of Galway, Galway, Ireland; 12 Department of Molecular and Cell Biology, University of California, Berkeley, CA USA; 13 Chan-Zuckerberg Biohub, San Francisco, CA USA

**Keywords:** Biodiversity, Phylogenetics, Evolutionary ecology

## Abstract

The evolutionary relationships among decapodiform lineages (cuttlefish and diverse types of squid) remain uncertain, with implications for the origin of internalized structures (for example, gladius, cuttlebone and coiled shell) derived from the ancestral chambered shell as well as the ecological shifts between the deep ocean and shallow coastal habitats. To address these questions, we adopted a phylogenomic approach that integrated three new high-quality genome sequences with available genomic and transcriptomic datasets. Our analyses support a novel topology that separates a clade of open-ocean lineages (Oegopsida and Spirulida, together Acorneata) from a clade comprising the remaining coastal and shallow-water orders (Sepiida, Myopsida, Idiosepiida and Sepiolida, together Corneata). Molecular clock estimates suggest a rapid cladogenesis of modern decapodiform orders in the deep open ocean during the mid-Cretaceous, consistent with fossil data. This early diversification set a ‘long fuse’ that led to the explosive radiation of squid and cuttlefish into coastal and shallow-water environments as they recovered from the Cretaceous–Palaeogene extinction event.

## Abstract (web summary)

Phylogenomic analyses of newly sequenced and published decapodiform cephalopods separate open-ocean and coastal shallow-water clades, following rapid cladogenesis from the mid-Cretaceous.

## Main

**Fig. 1:**
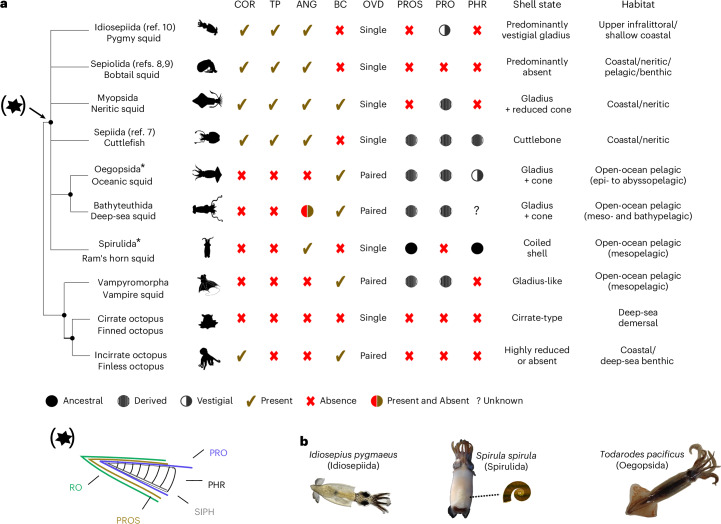
Evolution of key morphological traits and current phylogenetic context of decapodiform lineages. **a**, Representative morphological characters of decapodiform lineages mapped to an unresolved decapodiform tree. RO, rostrum; SIPH, siphuncle; PROS, primordial rostrum; PRO, proostracum; PHR, phragmocone; ANG, accessory nidamental gland; COR, cornea; TP, tentacle pockets; OVD, oviduct; BC, branchial canal. The asterisk next to Spirulida and Oegopsida indicates that these two orders appear as sister early branching clades in our phylogeny. References cited in brackets for Idiosepiida, Sepiolida and Sepiida refer to prior studies that place these lineages as the earliest diverging decapodiform. In Bathyteuthida, the ANG is present in one family but absent in another, as indicated by the mixed presence/absence code. PROS, PRO and PHR are marked as ‘derived’ or ‘vestigial’ in most taxa based on the presence of homologous structures (for example, cuttlebone in Sepiida) that originate from the chambered shell of ancestral decapodiform. In this context, derived refers to functionally elaborated structures that evolved from ancestral components, whereas vestigial refers to highly reduced, non-functional remnants (for example, septa-like elements of the phragmocone in Oegopsida gladius). Only Spirulida retains a fully mineralized, chambered form with two out of three ancestral components. The shell diagram represents ancestral decapodiform characters. Some of the morphological traits were previously reported by Lindgren et al.^14^. The habitat category provides a summarized classification; more detailed ecological distributions are shown in Supplementary Table 1. **b**, Species with newly sequenced genomes presented in this study. Shell illustration in **a** adapted from ref. ^142^ under a Creative Commons License CC BY 4.0. Silhouettes in **a** adapted from PhyloPic under a Creative Commons License CC0 1.0. Credit for *S. spirula* photo in **b**: Victor Tuset. Bathyteuthis abyssicola silhouette in **a** adapted from ref. ^143^, Neill.

Decapodiform cephalopods (cuttlefish and most types of squid) are a morphologically diverse clade of active molluscs that occupy ecological niches ranging from upper infralittoral zones to the deep sea (Fig. 1). While cuttlefish (order Sepiida) are uniquely characterized by their cuttlebone (an internal shell structure), true squid do not correspond to a single evolutionary lineage and are split into six orders whose common names (bobtail squid, pygmy squid, oceanic squid, neritic squid, deep-sea squid and ram’s horn squid) are based on body size, shape or ecological traits. Indeed, despite its name the so-called vampire squid *Vampyroteuthis infernalis* is actually an octopodiform cephalopod more closely related to octopuses than to true squid. Although resolving the relationships among the various squid and cuttlefish lineages is key to understanding the evolutionary origins of the extensive morphological diversity and ecological transitions within this group, these relationships remain unclear.

A hallmark of decapodiforms is the internal shell, a feature they share with their extinct sister group, the diplobelid belemnoids^1,2^ (Fig. 1). Indeed, among the few morphological features that retain clear lineage-specific signatures across decapodiforms (Fig. 1), the internal shell is perhaps the only one that still reflects a clear shared evolutionary origin despite its many modified forms^3^. Particularly enigmatic is the interpretation of the internal shell of the ram’s horn squid (order Spirulida), whose shell matrix proteins show only limited overlap with those of cuttlebones^4–6^.

Further long-standing questions concern the timing of the origin of decapodiform orders and their ensuing ecological diversification across oceanic and coastal habitats. Answers to these questions depend on the phylogenetic relationships among decapodiforms, yet analyses with different methodologies and datasets have not reached a consensus. Most molecular phylogenies placed coastal and shallow-water orders as the earliest branching decapodiform lineage, including cuttlefish (order Sepiida)^7^, bobtail squid (order Sepiolida)^8,9^ and pygmy squid (order Idiosepiida)^10^. Because belemnites (Belemnitida) were historically placed near the base of decapodiforms, their primarily coastal, shallow-epipelagic habitats^11^ were also used to argue for a coastal origin of the clade. Estimates for the origin of the crown group also vary considerably, from ~220 to ~107 Myr ago (Ma) (refs. ^7–9,12^).

These studies, albeit with different topologies, generally point to a coastal origin for the group and imply that the ancestor of modern decapodiforms lacked a primordial rostrum and phragmocone entirely, and possessed only a simplified proostracum or possibly lost these components entirely. Under this scenario, the cuttlebone of sepiids, the internal shell of spirulids and the gladius of oegopsid and myopsid squid represent convergent elaborations of internal shell structures from a simpler ancestral state^13^. Only the sister relationship between the oceanic squid Oegopsida and Bathyteuthida has remained stable across studies and is consistently supported by morphology, multiple molecular datasets and analyses^14–17^.

Palaeontological data raise the alternative possibility of a deep-sea origin for decapodiforms. The fossils *Groenlandibelus* (a stem spirulid) and *Longibelus* exhibit a combination of belemnoid and decabrachian features and share morphological characters suggesting that they inhabited deeper pelagic waters, approximately 100–250 m deeper than typical belemnite habitats, similar to those occupied by extant Spirulida (mesopelagic waters above the slope)^1^. Both taxa disappeared by the Maastrichtian during the Cretaceous–Palaeogene (K–Pg) boundary extinction. If *Longibelus* indeed represents a transitional form between diplobelid belemnoids and early decapodiforms, this would imply that decapodiforms originated from ancestors adapted to deeper oceanic open-ocean habitats during the Early Cretaceous rather than from coastal lineages. Supporting this scenario, a recent fossil study^18^ identified lower beaks of both oegopsids and myopsids in Late Cretaceous sediments, indicating that these crown decapodiform orders were already established in the mid-Cretaceous ~100 Ma, a period of sea level rise and stronger deoxygenation of shallow waters^19,20^. This interpretation, however, is inconsistent with the accepted hypothesis of a coastal origin for decapodiforms based on current phylogenetic analyses^7,10,12^.

Here we address these questions in decapodiform evolution with a phylogenomic approach. To aid in this analysis we present high-quality chromosome-scale genomes for the ram’s horn squid *Spirula spirula* and the tropical pygmy squid *Idiosepius pygmaeus*, which fill critical gaps in taxonomic representation, and add the Japanese flying squid *Todarodes pacificus* to include a second family within Oegopsida. Our analyses support an early split between open-ocean pelagic lineages, including the oceanic squid (Oegopsida) and ram’s horn squid (Spirulida), and a clade of coastal and shallow-water taxa comprising cuttlefish (Sepiida), neritic squid (Myopsida), pygmy squid (Idiosepiida) and bobtail squid (Sepiolida). Molecular clock analyses suggest that the origin of modern decapodiform orders occurred rapidly in the mid-Cretaceous, ~100 Ma. Given the absence of suitable shallow and coastal habitats at this time, we suggest that the cladogenesis of decapodiform orders occurred in the deep ocean. This early divergence lit a ‘long fuse’, and in most orders the radiation to coastal and shallow-water habitats occurred only after the K–Pg mass extinction, with implications for shell evolution, adaptive diversification and changes in genome architecture.

## Results and discussion

### Chromosome-scale genomes of previously unsampled decapodiform lineages

**Extended Data Fig. 1:**
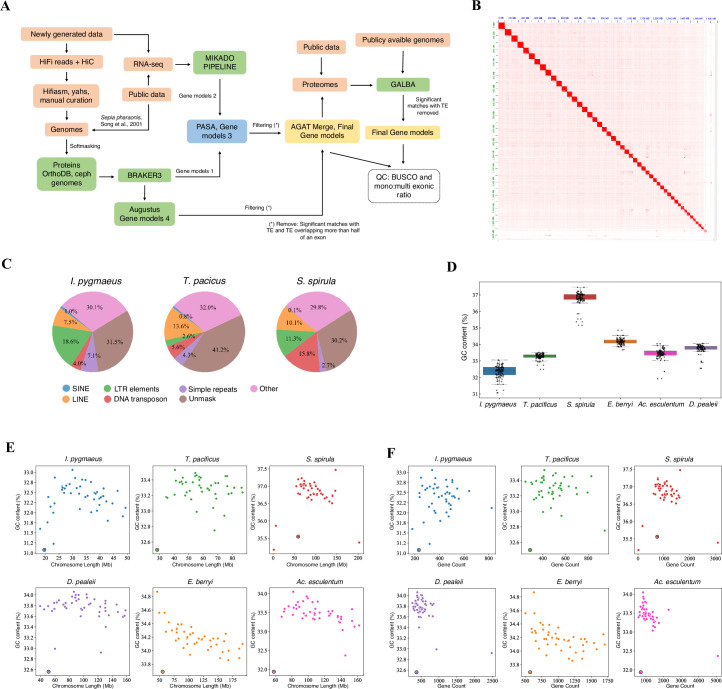
Overview of genome assembly, structure, and composition across newly sequenced cephalopod genomes. (**A**) Schematic pipeline for genome assembly and annotation. (**B**) Hi-C contact map of *Idiosepius pygmaeus* showing chromosome-scale scaffolding. (**C**) Repeat composition profiles across the newly sequenced genomes. (**D**) GC content distribution across chromosomes in the newly sequenced and reference decapodiform genomes. (**E**) Relationship between GC content and chromosome length in the newly sequenced and decapodiform genomes. (**F**) Relationship between GC content and gene count per chromosome in the newly sequenced and reference decapodiform genomes. The circularized dot in (**E**) and (**F**) indicates the sex chromosome.

We generated high-quality chromosome-scale genome assemblies for two previously unsampled lineages—the pygmy squid *Idiosepius pygmaeus* (order Idiosepiida) and the ram’s horn squid *Spirula spirula* (order Spirulida)—and for the Japanese flying squid *Todarodes pacificus* to expand representation within the Oegopsida (Methods, Supplementary Note 1 and Extended Data Fig. 1). All genome assemblies include a Z sex chromosome^21^. The haploid genome sizes vary from 1.63 gigabases (Gb) for *I. pygmaeus*, to 2.74 Gb for *T. pacificus* and 4.55 Gb for *S. spirula*, with scaffold (that is, chromosome) N50 lengths of 35.48 megabases (Mb), 62.62 Mb and 96 Mb, respectively (Supplementary Table 2 and 3). The genome of *I. pygmaeus* is the smallest reported to date among coleoid cephalopods (Supplementary Table 3). We annotated these genomes (Extended Data Fig. 1) and compared them with 11 other available genomes (Supplementary Table 2) to identify 13,815 gene families present in the majority of decapodiform genomes. Of these, 10,548, 12,193 and 11,202 included at least one gene from *I. pygmaeus*, *S. spirula* and *T. pacificus*, respectively. Gene model (BUSCO^22^) completeness was very high for *I. pygmaeus* (95.4%) and *T. pacificus* (96.9%), and relatively high for *S. spirula* (89.1%) compared with previously reported coleoid genomes (Supplementary Table 4).

Our newly sequenced genomes fill key phylogenetic gaps and provide representative assemblies for nearly all recognized decapodiform orders with the exception of Bathyteuthida, which is confidently sister to Oegopsida based on morphology and multiple molecular datasets and analyses^14–16^ (Fig. 1). Together with genomes generated by the Aquatic Symbiosis Genomics Project of the Wellcome Sanger Institute^23^ and publicly available datasets from other studies (Supplementary Table 2), this expanded genomic resource offers a robust framework to understand the evolution of decapodiform cephalopods. Our use of genome sequences (or a non-neural transcriptome in the case of *V. infernalis*) avoids possible effects of messenger RNA editing in transcriptomes from neural samples.

### Phylogenomics of decapodiform cephalopods

**Fig. 2:**
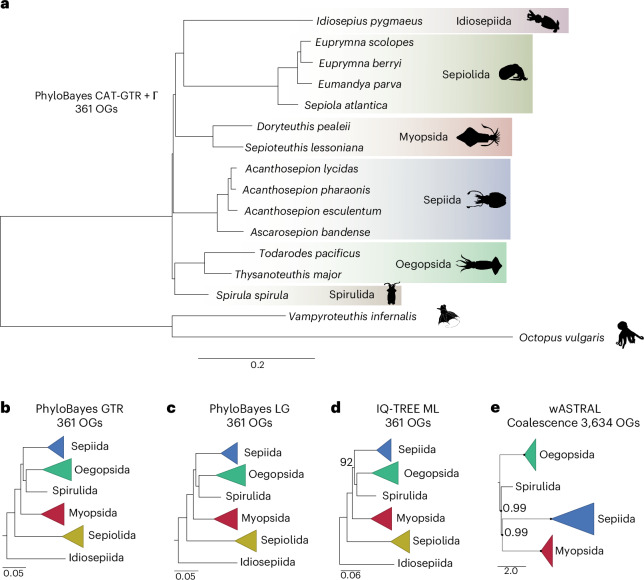
Phylogenetic relationships of decapodiform cephalopods inferred using different evolutionary models and gene subsets. **a**, The PhyloBayes tree reconstructed under the CAT-GTR + Γ model of evolution. **b**–**d**, Results of alternative models, tested and rejected in favour of CAT-GTR + Γ (Supplementary Note 2), including the PhyloBayes tree inferred using the GTR model (**b**), the LG model (**c**) and a ML tree (**d**). **e**, The species tree inferred with wASTRAL, with Idiosepiida and Sepiolida removed. Node support values are 1 for Bayesian PP in Bayesian trees, 100% bootstrap support in ML trees and 1 for local PP in wASTRAL trees, unless otherwise indicated. Silhouettes in **a** adapted from PhyloPic under a Creative Commons License CC0 1.0.

**Extended Data Fig. 2:**
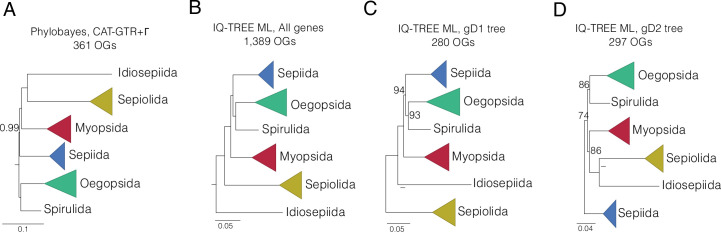
Bayesian and Maximum likelihood (ML) species trees and gene concordance based on different subsets of orthologous genes. (**A**) Phylobayes CAT-GTR + Γ tree inferred using *Ar. argo* as an additional outgroup. (**B**) ML tree topology inferred from the full dataset of 1,389 single-copy orthologous genes (OGs). (**C**) ML tree topology inferred from a subset of 280 OGs supporting an alternative topology consistent with gene discordance pattern 1 (gD1). (**D**) ML Tree topology inferred from a subset of 297 OGs supporting a different alternative topology consistent with gene discordance pattern 2 (gD2). Node support values are 1 for bayesian posterior probabilities Phylobayes tree, 100% bootstrap support in ML trees, unless otherwise indicated.

**Extended Data Fig. 3:**
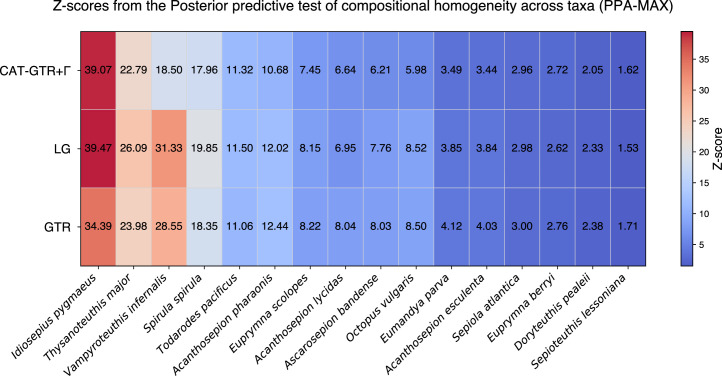
Posterior predictive test. Z-scores from compositional homogeneity across taxa.

With these data in hand, we examined the phylogenetic relationships from a phylogenomic perspective starting with a dataset of 1,398 single-copy orthologous genes (OGs) shared across 14 decapodiform genomes and two octopodiform outgroups (that is, the genome of *Octopus vulgaris*^24^ and a transcriptome from the vampire squid *Vampyroteuthis infernalis*; Methods) (Supplementary Table 2). Because phylogenetic inference can be sensitive to dataset composition and model assumptions^25^ (Supplementary Note 2 and Extended Data Fig. 2), we implemented additional filtering steps to improve the reliability of our analysis. We took advantage of our phylogenomic sampling (including multiple species within Oegopsida, Sepiolida, Myopsida and Sepiida) to further select single-copy orthologues with strong phylogenetic signal by removing genes whose maximum likelihood (ML) trees (1) showed highly supported violation of intra-lineage relationships^26^ and (2) had long terminal branches. We further restricted our attention to high-quality sequence alignments by filtering out genes with high saturation, multiple substitutions per site or low sequence diversity. These refinements yielded a final set of 361 high-quality OGs and a data matrix of 152,405 aligned sites, of which 46,084 were parsimony-informative. Bayesian phylogenetic analysis under the site-heterogeneous model CAT-GTR + Γ^27^ resulted in a phylogenetic tree with high posterior probabilities (PP) at each node (PP = 1; Fig. 2a). We tested and validated this result for modelling, long-branch, and incomplete lineage sorting artefacts, as well as robustness to outgroup choice (Fig. 2b–e, Methods, Supplementary Note 2, Supplementary Tables 5 and 6, and Extended Data Figs. 2 and 3).

Our phylogenomic analysis reveals a novel decapodiform topology (Fig. 2a) that separates open-ocean lineages (Oegopsida and Spirulida) from those inhabiting coastal and shallow environments (Idiosepiida, Sepiolida, Sepiida and Myopsida). This result further rejects Naef’s Teuthida concept^28^, which grouped myopsid and oegopsid squid into a single clade (Supplementary Note 2). The coastal and shallow-water clade in our phylogenetic tree is characterized by two shared morphological characters absent from the open-ocean clade: a cornea and tentacle pockets (Fig. 1). We designate this clade Corneata, with cornea and tentacle pockets as diagnostic characters (COR and TP in Fig. 1, respectively), and its sister clade Acorneata, which lacks these two traits (Supplementary Note 2). This clade definition is closely related to d’Orbigny’s (1841)^29^ original separation of ‘Myopsidès’ (closed eyes) and ‘Oigopsidès’ (open eyes), which was also based on cornea presence or absence and included most coastal and shallow-water decapodiform lineages within ‘Myopsidès’ (Supplementary Note 2). However, because Myopsida and Oegopsida are now used to refer to specific decapodiform orders, we propose the clade names Corneata and Acorneata to avoid nomenclatural conflict. (The alternative would be to use Myopsida and Oegopsida sensu d’Orbigny or sensu lato, taking care to differentiate the broader clades from orders with the same name.) The vampire squid outgroup also lacks a cornea, suggesting that this character is ancestral. Within the newly defined Corneata clade, Idiosepiida, Sepiolida and Myopsida form a monophyletic group that is sister to Sepiida. The Idiosepiida–Sepiolida group aligns with earlier results based on mitochondrial gene order^15^. The overall short internal branches suggest a rapid early cladogenesis of decapodiform orders whose timing we estimate below using a molecular clock.

**Extended Data Fig. 4:**
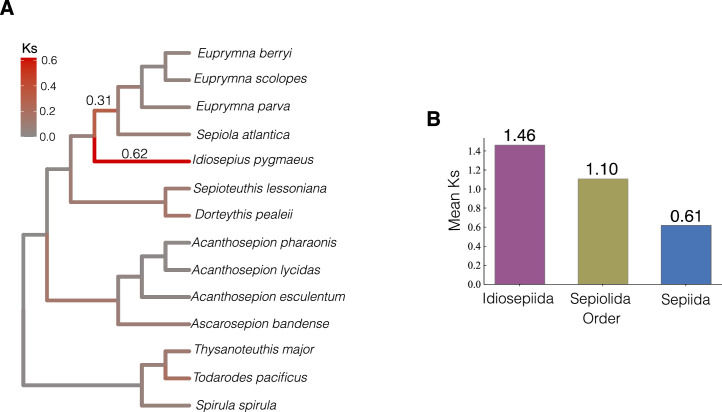
Accelerated evolution in Idiosepiida and Sepiolida. (**A**) Heatmap of synonymous substitution rates (Ks) across branches, estimated using the tree topology after the CAT-GTR + Γ model of evolution. (**B**) Pairwise Ks estimates of Idiosepiida, Sepiolida and Sepiida versus the rest of the lineages.

Our analyses consistently find that Idiosepiida, and to a lesser extent Sepiolida, are the most rapidly evolving decapodiform taxa. For example, Idiosepiida has the highest mean pairwise synonymous substitution rate^30^ to the other decapodiforms (Ks = 1.46), followed by Sepiolida (Ks = 1.10), and correspondingly long branch lengths^31^ in our phylogeny (Extended Data Fig. 4). For comparison, Sepiida has the lowest mean pairwise value (Ks = 0.61). This feature may explain the placement of Idiosepiida or Sepiolida as sister to other decapodiforms in earlier studies^9,10,32,33^, as a result of a long-branch attraction artefact. The long branch of Sepiolida is to some extent mitigated in our analysis by the inclusion of multiple bobtail squid genomes, although there is only a single member of Idiosepiida.

The short internal branches grouping Sepiida with Myopsida, Idiosepiida and Sepiolida (Fig. 2a) suggest a rapid cladogenesis of decapodiform orders. Because such rapid cladogenesis is particularly prone to incomplete lineage sorting, we generated an alternative species tree using weighted ASTRAL (wASTRAL)^34^, an approach that is statistically consistent under the multi-species coalescent model and better accounts for incomplete lineage sorting while further reducing the influence of low-support quartets and long terminal branches on species tree estimation. To minimize potential bias from compositionally heterogeneous and rapidly evolving taxa potentially subject to long-branch artefacts, we excluded Idiosepiida and Sepiolida, and retained single-copy OGs across the remaining species, which expanded our dataset to 3,634 OGs. The resulting wASTRAL ML tree recovered Sepiida and Myopsida as sister lineages with strong support (local PP = 0.99), consistent with their placement under the CAT-GTR + Γ model (Fig. 2e and Supplementary Note 2) and supporting the reliability of our phylogeny in accurately capturing the relationships of decapodiform lineages.

### Conserved macrosynteny among decapodiform lineages

**Fig. 3:**
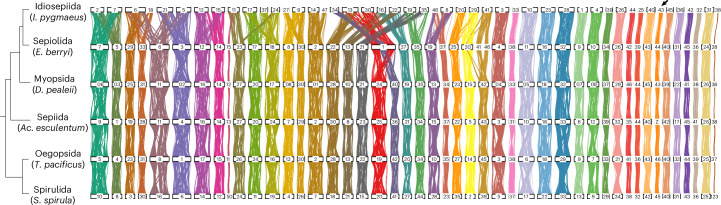
Conserved synteny across decapodiform lineages. Each vertical line represents one of 5,533 one-to-one OGs found in all six species. Lineage-specific rearrangements are evident in Sepiolida and Idiosepiida. See also Supplementary Note 3 and Extended Data Fig. 5. The arrow indicates the sex chromosome, present in all genome assemblies.

It has been suggested that the decapodiform ancestor had *n* = 46 pairs of chromosomes, based on the highly conserved karyotypes of Sepiida (*Acanthosepion esculentum* and *Acanthosepion* *lycidas*) and Myopsida (*Ssepioteuthis lessoniana*, *Uroteuthis (Photololigo) edulis* and *Heterololigo bleekeri*)^35,36^ and patterns of chromosome-scale conserved synteny observed between the genome sequences of *Doryteuthis pealeii* (Myopsida) and *Euprymna* spp. (Sepiolida)^37,38^. The most recent common ancestor of these orders, however, is not the decapodiform ancestor in our phylogeny. With our expanded taxon sampling we found that (1) Oegopsida and Spirulida also share this *n* = 46 karyotype with one-to-one chromosome correspondence with Myopsida and Sepiida; and (2) Sepiolida and Idiosepiida show independent chromosomal variation from this ancestral decapodiform karyotype (Fig. 3).

**Extended Data Fig. 5:**
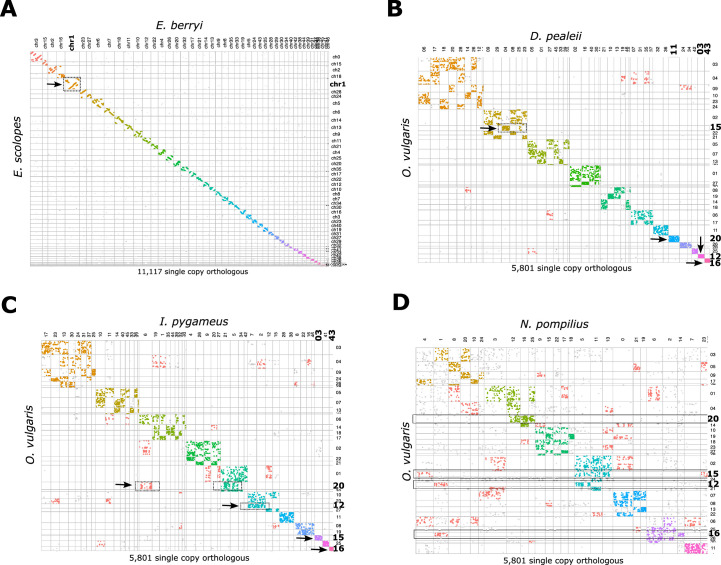
Comparison of chromosome architecture between cephalopod species pairs. (**A**) Macrosynteny conservation between *Euprymna berryi* (EBE) and *Euprymna scolopes* (ESC), showing a 1:1 correspondence among large scaffolds based on 11,117 shared single-copy orthologs. Dotted square boxes show the chromosome 1 of both species. (**B-D**) Oxford dot plots based on 5,801 shared single-copy orthologs between *Octopus vulgaris* and three other species: *Doryteuthis pealeii*, *Idiosepius pygmaeus*, and *Nautilus pompilius*. Chromosomes highlighted with dotted squares indicate syntenic regions used to polarize chromosomal changes and identify candidate markers for synapomorphic states in the decapodiform stem lineage.

Given our decapodiform phylogeny (Fig. 2), these observations are consistent with an *n* = 46 ancestor and derived fusions and rearrangements in the rapidly evolving Sepiolida and Idiosepiida (Fig. 3). The ‘near one-to-one’ correspondence previously described between *D. pealeii* and *Euprymna scolopes*^37^ is now clearly seen to involve a fusion of two ancestral decapodiform chromosomes (for example, DPE24 and DPE40) that form a single homologous chromosome in *Euprymna* (EBE1 and ESC1; Extended Data Fig. 5a) while still producing an *n* = 46 karyotype. This finding is consistent with a recent analysis of the *Sepia officinalis* genome^39^. Independently, the lineage of *I. pygmaeus* experienced multiple chromosomal rearrangements to produce an *n* = 47 karyotype. Notably, these two lineages also exhibit the highest rates of molecular evolution among decapodiforms (Extended Data Fig. 4).

Because these karyotypic deviations of *Euprymna* spp. and *I. pygmaeus* from the ancestral decapodiform state are not shared, they cannot be used as a synapomorphy for this group. Similarly, it is clear from chromosome-scale comparisons with the octopus and nautilus outgroups^37^ that the unique syntenic states seen in *Euprymna* spp. and *I. pygmaeus* are derived and cannot be ancestral to the *n* = 46 karyotype conserved in other decapodiforms (Supplementary Note 3 and Extended Data Fig. 5b–d). A recent draft assembly of the vampire squid genome *V. infernalis* shows greater syntenic similarity to decapodiforms than to octopodiforms chromosomes^40^, suggesting that the conserved decapodiform macrosynteny exemplified here by *T. pacificus*, *S. spirula*, *Ac. esculentum* and *D. pealeii* as shown in Fig. 3 carries patterns characteristic of early coleoid karyotype. Because the *Euprymna* and *I. pygmaeus* karyotypes are unique to their lineages, they unfortunately cannot be used as shared derived phylogenetic characters to directly support or reject other phylogenetic placements of Sepiolida and Idiosepiida.

### Decapodiforms arose rapidly in the mid-Cretaceous and later expanded to coastal habitats

**Fig. 4:**
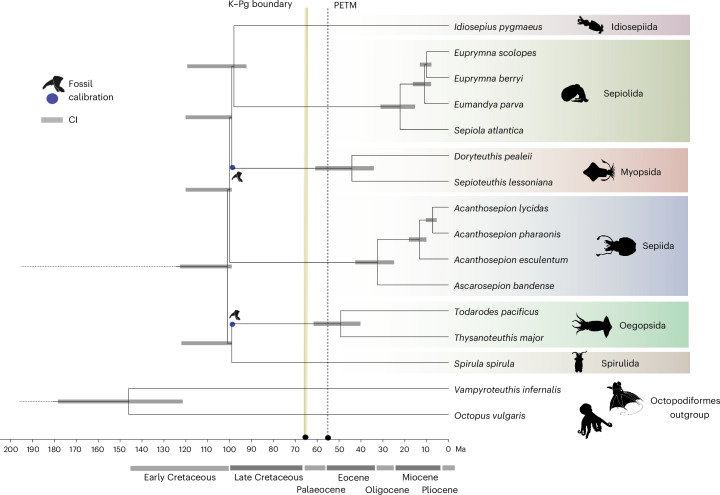
Time tree for decapodiform cephalopods. Divergence time estimate for crown group decapodiforms based on relaxed molecular clock, octopodiform–decapodiform divergence at ~240–245 Ma (refs. ^135,144,145^) and additional calibrations (Methods). PETM, Palaeocene–Eocene Thermal Maximum. All other silhouettes adapted under a Creative Commons License CC0 1.0.

We find a rapid origin of all crown group decapodiform orders with a point estimate of ~101 Ma (95% confidence interval (CI): 99–122.6) for the most recent common decapodiform ancestor, close to the Early–Late Cretaceous boundary (Fig. 4), based on a relaxed molecular clock analysis with several fossil calibrations^1,18^ (Methods and Supplementary Note 4). All major orders emerged rapidly at the end of the Early Cretaceous. These timings are broadly consistent with previous studies that used different inter-order topologies^8,9^, but are considerably younger than the Early Jurassic of ref. ^7^. An origin for crown group decapodiform orders around the Early–Late Cretaceous boundary is aligned with the concurrent extinction of belemnites^41^ and ammonites^42^.

Late Cretaceous sea levels were notably higher than the present day, with a concurrent stronger deoxygenation of shallow waters in epicontinental seas, and regional heterogenous deep-sea refugia^19,20,43,44^. Given the absence of suitable shallow and coastal habitats at this time, we suggest that the origination of order-level decapodiform lineages occurred in the open ocean. A rich diversity of fossil squid beaks has recently been described in carbonate concretions from Late Cretaceous shelf deposits in Japan from 70 to 100 Ma (ref. ^18^). These include stem loliginids from Myopsida and four fossil families with oegopsid-like beaks presumed to be stem oegopsids. Oegopsid beaks are, however, quite diverse^45^, and it is possible that these fossil beaks could be from stem decapodiforms (Supplementary Note 4). The co-occurrence of these oegopsid-like and stem-loliginid beaks in the same deposits is also consistent with early myopsids living in open waters. The open-ocean origin of decapodiforms suggested by our analysis implies that the mid-Cretaceous diversification lit a ‘long fuse’ that ultimately resulted in the convergent colonization of coastal environments by four related orders. The later radiations within these orders are notably more recent (see, for example, ref. ^8^) and postdate the K–Pg extinction event, which disrupted near-shore ecosystems and restructured marine ecosystems^46,47^. Their recovery and the subsequent development of more complex reef structures in the early Palaeogene may have supported this transition^48^.

Environmental stress associated with the K–Pg transition included a period of declining but persistent ocean acidification^49,50^ that probably contributed to the disappearance of many belemnite lineages due to the vulnerability of their aragonitic shells to ocean acidification^51^. The notable shell reductions in Idiosepiida and Sepiolida may have been an adaptation against acidification that allowed them to persist through a prolonged period of ecological restructuring of shallow coastal habitats. Fragmented, bottlenecked populations provide permissive conditions for underdominant chromosomal rearrangements to occur in these lineages; such rearrangements are generally suppressed in large well-mixed populations^52^ (Fig. 3). In contrast, large and well-mixed populations favour karyotype stasis, as observed in other decapodiform orders.

### Shell evolution

**Fig. 5:**
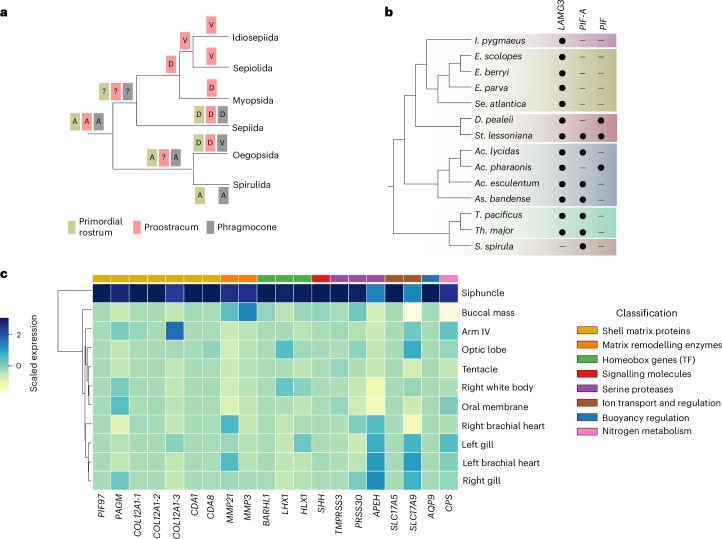
Evolution of shell and shell genes in decapodiforms. **a**, Parsimony reconstruction of three internal shell components across decapodiform lineages. Each box is annotated with its evolutionary state: A, ancestral; D, derived; V, vestigial. Trait absence indicates loss. Internal nodes marked with ? indicate uncertain character states. Traits generally follow a progression from ancestral to derived to vestigial/lost. **b**, Distribution of key biomineralization genes in decapodiforms. **c**, Tissue-specific expression of selected genes in the ram’s horn squid *Spirula spirula*. TF, transcription factors.

Our decapodiform phylogeny suggests that morphological transformations of the ancestral decapodiform shell reflect a progressive reduction followed by lineage-specific re-elaborations across extant lineages (Fig. 5a). Fossil evidence may also support this trajectory. For example, *Longibelus* exhibits a combination of belemnoid and decabrachian features. Based on seven key morphological characters, one study^1^ proposed that *Longibelus* may represent a transitional form between diplobelid belemnoids and early decapodiforms. In particular, *Longibelus* retains a primordial rostrum, a chambered phragmocone and a narrowed proostracum that is markedly reduced in width compared with non-diplobelid belemnoids, suggesting an early stage of shell simplification.

If *Longibelus* indeed represents a transitional form, the trajectory of internal shell reduction continues across all decapodiform lineages (Fig. 5a). In Spirulida, the phragmocone appears to have re-evolved as a ventrally coiled form, retaining a primordial rostrum, while the proostracum was lost. In Oegopsida, the phragmocone is vestigial, with septa-like remnants embedded within the posterior region of the gladius, often referred to as the rostrum or gladius cone^53^. These observations suggest that the common ancestor of Spirulida and Oegopsida (and possibly stem decapodiforms) probably retained a mineralized phragmocone and a primordial rostrum, which were subsequently modified in lineage-specific ways: retained and elaborated in Spirulida but reduced to vestigial structures in Oegopsida.

Among coastal and shallow-water lineages, Sepiida uniquely retained all three shell components, which were modified to become the cuttlebone, a flattened, chambered structure specialized for buoyancy control. In Myopsida, the phragmocone and primordial rostrum were lost, while the proostracum was retained and modified into a gladius in a manner similar to Oegopsida^53^, indicating that similar chitinous structures have arisen convergently. In Idiosepiida, the internal shell is reduced to a thin gladius that in most cases extends only partially along the dorsal mantle, while in Sepiolida most species lack an internal shell altogether, although some retain reduced gladius-like elements reminiscent of those in Idiosepiida^54,55^.

### Shell biomineralization gene evolution

To investigate molecular signatures of shell biomineralization we focused on homologues of three related families of von Willebrand factor type A and chitin-binding domain-containing genes required for shell biomineralization in molluscs^56^ (Supplementary Note 5 and Fig. 5b). These include the acidic matrix protein *Pinctada fucata* 97 KDa (*PIF97*) and a distant paralogue initially thought to represent the ancestral form of *PIF97* (referred to here as *PIF-A*^56–58^), both of which contribute to more rigid shell elements in bivalves^59,60^; *LAMG3*, which is expressed in the chalky layer of bivalves, a porous and flexible mineralized region with a key role in cementation and attachment to uneven substrates^57,61^; and the blue mussel shell protein (*BMSP*), which is functionally similar to *PIF97* but is characterized by expanded von Willebrand factor A domains^62^. While *PIF* homologues are present in nearly all decapodiform lineages, the clade comprising Sepiolida and Idiosepiida appears to have lost both *PIF97* and *PIF-A*, and *St. lessoniana* uniquely retains both paralogues. All lineages, except *S. spirula*, retain the *LAMG3* gene. This pattern suggests that *LAMG3* alone may retain a basic structural role in shell formation of decapodiforms even in highly reduced forms. We speculate that the short, thin and flexible gladius observed in some species may mirror the mechanical function of the chalky layer in bivalves. Additional data and functional studies from species with rudimentary gladii in both lineages are needed to confirm this hypothesis.

The presence of both *PIF97/PIF-A* and *LAMG3* broadly across squid and cuttlefish suggests that these proteins support the development of more rigid and complex structures such as the gladius or cuttlebone. A notable exception to this pattern is the absence of *LAMG3* in *S. spirula*, which suggests divergent regulation of shell formation in this species relative to other decapodiforms. To investigate this further, we performed differential gene expression analysis in *S. spirula* to identify genes specifically expressed in its siphuncle, a structural component of the chambered internal shell involved in buoyancy regulation. Because of its anatomical and functional link to the internal shell, the siphuncle provides a valuable window into the ancestral decapodiform shell physiology.

By comparative transcriptomics we identified 235 siphuncle-specific genes in *S. spirula*, including 78 with recognizable homology with known proteins in other molluscs and sometimes more broadly among metazoans (Supplementary Table 8). These include many known biomineralization toolkit genes in molluscs^63^ as well as genes associated with gas transport and ion exchange. Notably, we found *PIF97* to be uniquely expressed in the siphuncle, suggesting a role in facilitating calcium carbonate deposition within the chambered structure (Fig. 5c). This is further supported by the expression of the phosphoacetylglucosamine mutase (*PAGM*) gene (Fig. 5c), an essential enzyme in chitin biosynthesis, indicating that the siphuncle helps maintain shell integrity through an active chitin-based framework in the ram’s horn squid.

In addition to *PIF97* and *PAGM*, we identified *S. spirula* siphuncle expressed genes associated with collagen stabilization, matrix remodelling, transcriptional regulation and signalling, including several previously detected in the *S. spirula* shell proteome^5^ (Fig. 5c, Supplementary Table 8 and Supplementary Note 6). We also found genes supporting roles in nitrogen metabolism, pH homeostasis and fluid regulation (Supplementary Note 6). Overall, our findings suggest that the siphuncle plays a broader physiological role beyond gas exchange, highlighting its unique regulatory function in shell maintenance, matrix remodelling and chamber fluid regulation, distinguishing it from internal shell derivatives (for example, cuttlebone, gladius) in other extant decapodiforms. This function may have contributed to the survival of *S. spirula*, while other similarly shelled lineages went extinct.

### Adaptive evolution and gene family expansion in decapodiforms

In light of the morphological and physiological adaptations within decapodiforms, we sought signatures of positive selection among decapodiform clades and lineages (Methods), recognizing that our inferences are limited to the species analysed here. We identified 32 genes with such significant signatures in ‘open-ocean’, ‘coastal plus shallow’ and ‘Idiosepiida plus Sepiolida’ clades and 305 genes in individual lineages (Supplementary Table 9). Here we highlight three examples with plausible links to ecological adaptations in specific clades in light of the deep-water origins of decapodiforms discussed above.

**Extended Data Fig. 6:**
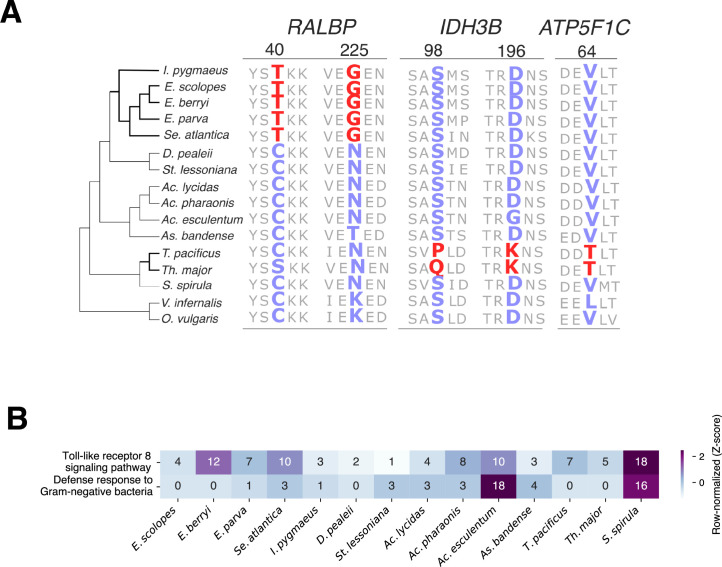
Adaptive evolution and gene family expansion in selected decapodiform lineages. (**A**) Signatures of positive selection in three target genes across Idiosepiida, Sepiolida, and Oegopsida. (**B**) Gene family expansion of immune-related genes in the ram’s horn squid *S. spirula*.

On the branch leading to the coastal shallow-water Idiosepiida and Sepiolida clade we found evidence for positive selection in the retinal-binding protein (*RALBP*) gene (Omega (*ω*) = 29.88) at two residues: C40T (Bayes empirical Bayes (BEB) posterior probabilities = 0.98) located within the CRAL-TRIO lipid-binding domain where retinal binds; and N225G, (BEB = 0.969) in the GOLD domain involved in protein trafficking^64^ (Extended Data Fig. 6a). *RALBP* was first functionally characterized in *T. pacificus*^65^, where it was shown to transport retinal molecules between photopigments within photoreceptor cells, maintaining the retinal recycling process required during light detection and ensuring sustained visual processing^66^. *RALBP* is expressed in the light organ and eye of the Hawaiian bobtail squid *E. scolopes*^67^, and is highly expressed in the eyes of other molluscs such as the Pacific abalone *Haliotis discus hannai*^68^ and the chiton *Leptochiton asellus*^69^. We speculate that the substitutions in *RALBP* under positive selection in the Idiosepiida–Sepiolida clade may reflect adaptive modifications in retinal processing efficiency in more light-variable shallow coastal habitats relative to the ancestral dim-light deep-water environment where decapodiforms arose.

On the branch leading to the open-water Oegopsida we found evidence for positive selection in two enzymes involved in mitochondrial adenosine triphosphate (ATP) production: ATP synthase subunit gamma (*ATP5F1C*) (*ω* = 15.60) and isocitrate dehydrogenase subunit beta (*IDH3B*) (*ω* = 6.39). ATP synthesis occurs through the rotation of the ATP synthase complex, with the gamma subunit acting as the rotor that drives conformational changes in the catalytic nucleotide-binding sites of the β-subunits, leading to the formation and release of ATP^70^. We detected positive selection at a single residue in *ATP5F1C*, V64T (BEB = 0.992), possibly improving coupling efficiency under high protein flux (Extended Data Fig. 6a). *IDH* catalyses the conversion of isocitrate to α-ketoglutarate, generating NADH, which donates electrons to the electron transport chain, establishing the proton gradient that drives ATP synthesis^71–73^. *IDH3* exhibited significant positive selection at two sites: S98P/Q (BEB = 0.984) and D196K (BEB = 0.984) (Extended Data Fig. 6a). At position 98, *Thysanoteuthis major* possesses glutamine (Q), while *T. pacificus* presents proline (P), both derived from an ancestral serine (S). These substitutions may represent a trajectory of increasing structural specialization (Supplementary Note 7). Changes to these key energy-generating enzymes may reflect elevated metabolic demands associated with fast, sustained swimming in pelagic environments, although we caution that our analysis includes only two representative species from two families from a highly diverse clade.

Finally, we investigated the functional consequences of gene family expansions and contractions during decapodiform evolution (Methods). We found a modest gene gain or loss rate (*λ*) of 0.0025 Myr^−1^, corresponding to ~3,000 gene gains or losses since the origin of crown decapodiforms ~101 Ma. In several cases, gene family expansions were associated with immune response, including significant expansion in Spirulida of (1) the Toll-like receptor 8 (TLR8) signalling pathway, which is involved in pathogen recognition and immune activation and is highly diversified in metazoans and especially bivalves^74–76^; and (2) Gene Ontology (GO) terms linked to innate immune defence against Gram-negative bacteria (Methods and Extended Data Fig. 6b). These expansions in *S. spirula* relative to other decapodiforms may reflect a specialized immune repertoire that regulates interactions with both pathogens and microorganisms in its unique deep-sea habitat^77^.

## Conclusions

Here we present an updated framework for decapodiform evolution made possible by three additional genome sequences, including previously unrepresented pygmy squid and ram’s horn squid lineages, and application of site-heterogeneous models for sequence evolution. Our decapodiform phylogeny shows an early split between open-ocean lineages and a coastal and shallow-water clade, occurring in the mid-Cretaceous ~101 Ma, followed by a rapid cladogenesis of decapodiform orders. Although the branching order of our phylogeny differs, the timing of these splits to the mid-Cretaceous is consistent with other molecular clock analyses^8,9^. The absence of suitable oxygenated near-shore habitats at this time of rising sea levels and the presence of patchy deep-sea refugia during anoxic events^44^, along with recent fossil evidence^18^, suggests that the early crown decapodiform lineages lived and diversified in the deep, open ocean, a habit preserved in contemporary oegopsids and spirulids. It is only later, when shallow-water environments recovered after the K–Pg extinction, that the other decapodiform lineages radiated into their contemporary coastal habitats in response to these new ecological opportunities. Similar evolutionary patterns of persistence across the K–Pg boundary from deep or open-ocean ancestry have been observed in other marine animal groups including elasmobranchs^78^, spiny-finned fishes^79,80^, deep-water teleosts^81,82^, pelagic Scombriformes^83^, several widely distributed ammonites and nektobenthic nautilids (occupying seafloor depths to ~600 m)^42^.

The delayed expansion across multiple decapodiform orders resembles the ‘long fuse’ model developed for placental mammal evolution^84,85^ in the sense that the early splits between decapodiform orders preceded the radiation of these lineages into newly available coastal and shallow-water habitats by tens of millions of years. The mesopelagic spirulids, represented by a single extant genus, are an exception, diverging from oegopsids in the Late Cretaceous but also retaining the ancestral open-ocean lifestyle. This ‘long fuse’ model for decapodiform cephalopod evolution is supported not only by phylogenomics and the fossil record, but also by evidence for adaptive evolution in Idiosepiida and Sepiolida in genes plausibly reflecting molecular adaptations to increased light exposure in coastal shallow environments. Our study suggests that greater family, generic and species richness of decapodiform cephalopods compared with octopodiforms, and their morphological and physiological adaptations to diverse lifestyles^86^, is the end result of this ‘long fuse’ that ultimately resulted in the convergent colonization of coastal environments from their open-ocean ancestors.

## Methods

### Animal collection

Three female pygmy squid *Idiosepius pygmaeu*s, each approximately of 1 cm in dorsal mantle length (DML), were collected off the coast of Seragaki in Onna, Okinawa, by the Okinawa Institute of Science and Technology (OIST) Cephalopod Team in November 2023. Species identity was confirmed by BLAST search of the mitochondrial contig (obtained post-assembly; see below) against the National Center for Biotechnology Information (NCBI) database. A young male Japanese common squid *Todarodes pacificus* of 17 cm DML was also collected in Hakodate, Japan, in October 2021. In addition, two young individuals of the ram’s horn squid *Spirula spirula* of around 2 cm DML were collected off the Canary Islands in February 2023 by A.B. onboard the RV *Sarmiento de Gamboa*. The animals were collected at mesopelagic depths and placed at −80 °C right after they reached the surface.

The pygmy squid and Japanese flying squid were acclimated in seawater aquaria at 21 °C for 2–4 h. After acclimation, individuals were euthanized using a solution of seawater with ethanol, which was gradually increased from 1% to 3%, following ethical and welfare recommendations for cephalopod research^87^, prior to dissection of multiple organs (Supplementary Table 10). Organs were stored immediately at −80 °C. For the ram’s horn squid, both individuals were immediately placed at −80 °C on board, and subsequently shipped to OIST for dissection. Organs of the ram’s horn squid were dissected in RNAlater-ICE (Invitrogen) using standard protocols.

### Genome sequencing, assembly and completeness

For each of our three species, high molecular weight DNA was isolated from the mantle muscle of a single individual with the skin removed using the PacBio Nanoind tissue kit (PacBio, 102-302-100). Genomic DNA was shared using Megaruptor 3. Fragment sizes and concentration of DNA were assessed using a Femto Pulse System (Agilent Technologies). HiFi SMRTbell libraries were prepared using a SMRTbell Prep Kit 3.0 (PacBio, 102-182-700) following the manufacturer’s protocol. Library size selection was performed using the BluePippin system (SageScience) with High Pass Plus cassettes (BPLUS10). Sequencing was performed under circular consensus sequence mode using the PacBio Sequel IIe HiFi and Revio (Pacific Biosciences).

To reach chromosome scale, we generated proximity ligation Hi-C libraries using the Omni-C kit from Dovetail Genomics, following the manufacturer’s protocol. These libraries were sequenced on the Illumina NovaSeq6000 platform to achieve a final 15–30X coverage based on the unique read pairs and preliminary estimations of genome size using contig-level assemblies (see below). For *T. pacificus*, both PacBio and Omni-C reads were obtained from the same individual. However, for *I. pygmaeus* and *S. spirula*, we used different individuals due to the small size of the specimens.

HiFi reads were used to generate the primary assemblies for *I. pygmaeus* and *S. spirula* using hifiasm v0.19.6-r595^88^. For *T. pacificus*, a haplotype-resolved assembly was generated using hifiasm with the integration of Hi-C data. Duplicate regions in the primary assemblies were removed using Purge Haplotigs v1.1.3^89^.

Hi-C valid read pairs were obtained by aligning reads to the assemblies using BWA-MEM v0.7.17-r1188^90^, followed by parsing using pairtools v1.0.2^91^ allowing minimum mapping quality of Q30. These valid read pairs were used to anchor and orient contigs into chromosome assemblies using YaHS v1.2^92^. The resulting assemblies were manually curated using Chromap v0.2.5-r473^93^, the Juicebox Assembly tool v2.20^94,95^ and Artisanal (https://bitbucket.org/bredeson/artisanal). Assembly completeness was assessed using BUSCO v5.5.0^22^ genome mode, with miniprot as gene mapper.

### Annotation of transposable elements

Custom libraries of transposable elements (TE) were identified with RepeatModeler v2.0.5^96^, which employs RECON v1.0.8, RepeatScout v1.0.7 and LTR_retriever/LTRHarvest v2.9.0. These libraries were used to annotate and mask the genomes using RepeatMasker v4.1.5. For *S. spirula*, LTR_retriever/LTRHarvest within the RepearModeler pipeline did not complete, probably because of the larger genome size and abundance of simple repeats. To overcome this, TE annotation was divided into two parts: (1) simple repeats were hard-masked, and LTR_retriever/LTR_harvest v2.9.9^97^ was run on the hard-masked genome; (2) the libraries generated by RepeatModeler (excluding LTRharvest) were combined with those from LTR_retriever/LTR_harvest. The genome was then annotated and masked using RepeatMasker v4.1.5 using the combined library.

### Transcriptome and protein-coding annotation

Total RNA was isolated from multiple organs (Supplementary Table 10) using the RNeasy Micro and Mini kits (Qiagen) following the manufacturer’s instructions. Strand-specific libraries were prepared using the TruSeq RNA library kit and sequenced using lanes of Illumina NovaSeq6000 system in 2 × 150 bp paired-end mode. Raw reads were trimmed for quality and adaptor sequences using Fastp v0.23.2^98^, mapped to the genomes with STAR v2.7.11a^99^ and Hisat2 v2.2.1^100^, and assembled using StringTie v2.2.1^101^. Moreover, reads were assembled using both genome-guided and de novo using Trinity v2.15.1^102^ followed by the alignment of transcripts to the genome using GMAP v2023-12-01^103^. In addition, a set of valid junctions were generated using Portcullis v1.2.4^104^ based on the STAR bam files merged with Samtools v1.12^105^. These splice junctions along with the transcriptome evidence were processed through the Mikado v2.3.4 pipeline^106^ to generate the set of best protein-coding transcripts for the species.

Gene models were generated using a combination of Mikado transcripts and BRAKER v3.06^107^ predictions. BRAKER3 was used to produce a preliminary set of gene models by integrating evidence from both protein sequences and RNA sequencing (RNA-seq) splice alignments. The protein sequences included the metazoan protein set from the OrthoDB v11 database^108^ and publicly available gene models from multiple cephalopod species^24,37,38,109,110^. RNA-seq splice alignments were obtained from the Portcullis output. The resulting gene models were refined by incorporating the Mikado transcripts using two rounds of the PASA v2.5.3^111^ annotation updates. Predictions with high similarity with TE from the RepeatPeps.lib database were removed using a BLASTp from Diamond v2.1.8.162^112^ with a 1 × 10^−25^ e-value cutoff. Additionally, gene models with less than half of their exons overlapping repeats were retained. Finally, mono-exonic genes were searched against the Pfam HMM database using PfamScan v1.6, and those without Pfam hits were removed.

During gene annotation, additional Hi-C reads were generated for *I. pygmaeus* and *S. spirula* to increase coverage and improve chromosomal assembly. This enabled a final round of genome refinement using Juicebox and Artisanal, as initial curation left some residual duplication in the primary assemblies. To ensure that the improvements in the final assemblies were accurately reflected in the gene annotations, we used Liftoff v1.6.3^113^ to transfer the previously generated gene models to the updated genome assemblies with parameters ‘-a 1 -s 1’. Gene model completeness was assessed using BUSCO v5.5.0 with miniprot as gene mapper with the metazoan dataset^22^.

### Protein-coding annotation of publicly available genomes

Several additional decapodiform genomes were downloaded from the NCBI database (Supplementary Table 2). Six of these genomes were deposited by the Aquatic Symbiosis Genomics Project from the Wellcome Sanger Institute and the other six belong to previously published studies^24,37,38,114–116^. Owing to the lack of RNA-seq data from multiple organs for these species, we leveraged well-annotated gene models from closely related species, which were predicted using RNA-seq splice alignments and protein evidence. The additional genomes were annotated using GALBA v1.0.10^117^ using the gene models from closely related species as references. For species in the order Sepiida, the publicly available genome of *Acanthosepion pharaonis* from ref. ^114^ was re-annotated using a combination of Mikado and BRAKER3, as described above, to reduce duplication observed in their original gene model predictions. The refined gene models for *Ac. pharaonis* were subsequently used to annotate other species from the order Sepiida. Finally, genes with significant match to TE in the RepeatPeps.lib database were removed, and the completeness of the gene models was evaluated using BUSCO protein mode.

### Functional gene annotation

We selected the longest isoform per gene and performed functional annotation using BLASTp from Diamond v2.1.8.162 to search against the SWISS-PROT database, applying a cutoff e-value of 1 × 10^−25^. Additionally, we used InterProScan v5.71-102^118^ and eggnog-mapper v2.1.12^119^ to extract additional domain annotation and GO terms from genes.

### *Vampyroteuthis infernalis* transcriptome

Raw RNA-seq reads from the arm tip of *Vampyroteuthis infernalis* were downloaded from NCBI (SRR5527416)^120^. Good quality reads were identified with Fastp, followed by assembly with Trinity. To retain only the most representative transcript per gene, the script filter_low_expr_transcripts.pl from Trinity was used, retaining the isoform with the highest expression levels. Gene-level abundance was calculated using the script align_and_estimate_abundance.pl from Trinity, with Kallisto^121^ as the quantification method, and isoforms with expression levels below two transcripts per million were excluded. Finally, protein-coding genes were predicted using TransDecoder v5.5.0 (https://github.com/TransDecoder/TransDecoder).

### Orthology inference and gene family analysis

Peptide sequences were obtained from a broad sampling of taxa that includes three newly sequenced decapodiform genomes from our study, 11 publicly available decapodiform genomes, and two octopodiform outgroups with both genome and transcriptome data available (Supplementary Table 2). The newly sequenced genomes allowed us to include at least one species from almost every decapodiform order. The only missing order is Bathyteuthida, whose phylogenetic position is well supported as sister to Oegopsida and it is consistent across various previous studies. Orthology reconstruction and gene family analysis were performed using Orthofinder v2.5.4^122^ using the longest protein sequence per locus for each species with an annotated genome.

### Orthology filtering, supermatrix generation and phylogenetic analysis

We filtered single-copy OGs present in all the taxa. Each OG set was aligned using MAFFT v7.4.1^123^ and regions with poor alignments were removed using Gblocks v0.91b^124^, excluding positions where gaps were present in half or more of the total taxa sequences. We generated ML trees for these OGs using two approaches: first, by creating a concatenated dataset of all OGs; and second, by generating individual gene trees for each OG. The latter approach was used to evaluate the agreement of the evolutionary history of each OG with the ML species tree using the gene concordance factor^125^. ML trees were constructed using IQ-TREE v2.3.5^126^ with ModelFinder^127^ to determine the best-fitting model for each alignment. Branch support was assessed using 1,000 ultrafast bootstrap replicates^128^. Orthogroups falling into two gene discordance factor classes (gD1, 280 OGs) and (gD2, 297 OGs) were used to create two additional datasets to generate alternative tree topologies, and to investigate phylogenetic conflicts.

We generated another dataset focusing on the best-quality genes by minimizing potential contamination and enhancing the phylogenetic signal. To achieve this, we excluded OGs having trees with long terminal branches, specifically those where any branch was 20 times or longer than the median branch length of the entire tree, except for branches belonging to the outgroup. Additionally, OGs whose trees violated well-defined clades were removed using Clan_Check^26^. Clan_Check was run under the assumption that species within each of the orders Oegopsida, Sepiida, Myopsida and Sepiolida should always form monophyletic clades. Gene trees that support the monophyly assumption were retained. Gene trees that violated this assumption were evaluated based on their bootstrap support. Genes that violate clade monophyly with strong bootstrap support (more than 50%) were excluded, because the strong support suggests the violations probably reflect paralogy or noise signal. Conversely, genes whose trees only weakly violate the monophyly assumption (bootstrap support of less than 50%) were retained, because the low support suggests that the violations might be due to weak phylogenetic signal rather than true discordance. Finally, we reduced the inclusion of OGs with saturated sites and low diversity by calculating regression slopes for each gene. These slopes were estimated as the linear regression coefficient between the ML pairwise genetic distance files generated by IQ-TREE, and percentage of identity values estimated using BLASTp from Diamond. Comparisons involving outgroup taxa were excluded, and OGs with the top 50% lowest regression slopes, indicating minimal saturation and greater diversity, were concatenated. Phylogenetic reconstruction of this final gene set was carried out in Phylobayes-MPI v.1.8^27^ under the site homogeneous models LG and GTR, and the site-heterogeneous model CAT-GTR along with discrete gamma distribution of four categories (CAT-GTR + Γ). We ran two independent chains for each model until convergence, assessed using Phylobayes’s bpcomp (requiring a maximum difference, maxdiff, <0.3) and tracecomp (requiring a relative difference, rel_diff, <0.3 and effective sample size, ESS, >50), applying a 15% burn-in. The LG and GTR models reached convergence within a relatively short number of generations. For the CAT-GTR + Γ model, we ran two chains for over 6,000 generations, achieving convergence with maxdiff = 0, a minimum ESS of 79 and rel_diff = 0.28.

### Model fit assessment

To evaluate the fit of CAT-GTR + Γ, LG and GTR models to the data, we conducted posterior predictive tests of amino acid diversity (PPA-DIV, readbp_mpi -div)^129^ and compositional homogeneity across taxa (PPA-MAX, readbp_mpi -comp)^130^, as implemented in PhyloBayes-MPI. Model fit was assessed at 10,000 generations with a fixed topology, using the absolute *Z*-scores, with smaller values indicating a better fit of the model.

### Evaluating and validating phylogenetic topologies

Preliminary studies and our findings based on ML trees suggest that either Idiosepiida, Sepiolida or Sepiida diverged earlier than other clades. To evaluate whether their placement is influenced by accelerated molecular evolution, we estimated the synonymous substitution rates (Ks) using two different approaches. First, we applied the free-ratio model in the codeml program implemented in PAML 4.9^31^ to estimate branch-specific synonymous substitution rates under a fixed topology inferred using the CAT-GTR + Γ model. For this analysis, we used a concatenated codon alignment of high-quality 361 single-copy orthologues used to generate our CAT-GTR + Γ based phylogenetic tree. Second, we calculated pairwise Ks values between each species of the target lineages (Idiosepiida, Sepiolida and Sepiida) versus species within Oegopsida, Spirulida and Myopsida. Orthofinder was run for each species pair, and OGs were aligned using MAFFT and back-translated to codon-based alignments. These rates were calculated using the Yang and Nielsen method^30^ implemented in the YN00 program from PAML. Ks values larger than 98 or less than 0.01 were discarded.

To further assess whether the species tree is influenced by incomplete lineage sorting, we generated a phylogenetic tree using coalescence models implemented in wASTRAL^131^ based on ML trees generated by IQ-TREE. We used the hybrid mode of wASTRAL, which reduces the impact of both quartets with low support and long terminal branches. For this analysis we excluded Idiosepiida and Sepiolida due to their accelerated evolution and potential long-branch attraction, which ASTRAL does not model accurately. OGs were identified using Orthofinder, aligned with MAFFT, and gaps were removed using Gblocks, as previously described. Tree support was evaluated using local PP (-u 2), with 16 rounds of placements to optimize the exploration of species positions on the tree and minimize quartet distance to the gene trees.

### Macrosynteny analysis

One representative species from each major decapodiform order was selected for macrosynteny analysis. This included *D. pealeii* from Myopsida, *Ac. esculentum* from Sepiida, *E. berryi* and *E. scolopes* from Sepiolida, as well as the three newly sequenced genomes reported in this study (*T. pacificus* from Oegopsida, *S. spirula* from Spirulida and *I. pygmaeus* from Idiosepiida). To polarize syntenic changes, we also included *Octopus vulgaris* and *Nautilus pompilius*. Depending on the analysis, different combinations of species were used to assess syntenic conservation (for example, comparisons restricted to decapodiforms, or pairwise comparisons). Single-copy OGs were identified using OrthoFinder, and significant macrosynteny blocks were estimated using Fisher’s exact test as described in ref. ^132^ and implemented in MacrosyntR v2.19^133^. Results were visualized with chord diagrams and Oxford dot plots generated within MacrosyntR.

### Molecular dating

We estimated a time-calibrated tree using the least-square dating method (LSD2)^134^ that applies a lognormal relaxed clock to the branch lengths. We used the tree estimated with the CAT-GTR + Γ model with a lognormal relaxed clock of 0.2 standard deviation. Confidence intervals for divergence times were estimated using 1,000 simulated trees. We set the root age to 240*–*245 Myr based on the fossil record of *Germanoteuthis donai*, and oldest stem octopodiform from the Triassic Ladinian and Asinian^135^. We considered the fossil of lower beaks identified by Ikegami et al.^18^ to oegopsids and myopsids as proposed, and applied corresponding calibrations at the early Cenomanian requiring both clades to be older than 99 Myr. The myopsid calibration was placed on the node representing the most recent common ancestor of Myopsida, Idiosepiida and Sepiolida, corresponding to the branch leading to Myopsida in our phylogeny. Similarly, the oegopsid calibration was placed on the most recent common ancestor of Oegopsida and Spirulida, corresponding to the branch leading to Oegopsida.

### Gene family expansion and contraction, and functional enrichment

We used the Computational Analysis of gene Family Evolution (CAFE) v5.1^136^ to identify gene families with significant expansions and contractions across our lineages, employing the Among Family Rate Variation (AFRV) model. Gene families were obtained from the phylogenetic hierarchical orthologous groups (HOGs) generated by OrthoFinder, using a tree topology inferred with the CAT-GTR + Γ model, and a time-calibrated tree estimated during the divergence time estimation. We included 11,910 HOGs by requiring at least one *O. vulgaris* gene per group, allowing *V. infernalis* to be missing due to its muscle transcriptome origin. An initial filtering of these HOGs having gene counts between 1 and 50 across all species was used to estimate both lambda and alpha parameters with two gamma rate categories. Using this filtered HOGs dataset, we estimated both lambda and alpha parameters with two gamma rate categories. Finally, CAFE was run again with fixed lambda and alpha values, retaining HOGs with gene counts between 1 and 100 across species. We retrieved gene families with significant changes using a *P* value of 0.05.

To assess the functional relevance of these gene families, we performed GO enrichment analysis using the TopGO v.2.54 R package^137^ with two-sided Fisher’s exact tests. We first minimize the impact of stochastic variation within each gene family (probably arising from incomplete annotations), by applying a gene count difference threshold: for expansions, we retained families with an increase of more than 10 gene copies; for contractions, we required a loss of at least 10 copies, which yielded 231 expanded and 11 contracted families. Second, we manually inspected the gene count matrix and selected HOGs where the expansion or contraction was specific to a target lineage, allowing at most one species outside that lineage to show a similarly high or low gene count. We selected one representative species per target lineage within each selected gene family and retrieved its GO annotation from eggNOG-mapper. GO terms associated with genes from each family were propagated to the level of the entire family as representative annotations. For enrichment analysis, the selected expanded or contracted families were used as the foreground set, while all other gene families served as the background. We focused on GO terms from the Biological Process domain and retained only those with uncorrected *P* values < 0.01.

### Adaptive evolution

We estimate positive selection using the rate of non-synonymous substitution (Ka) to synonymous substitution (Ks) (Ka/Ks = *ω*), with positive selection indicated as *ω* > 1. We used the optimize branch-site model^138^ implemented in codeml from PAML 4.9^139^ setting different lineages (independent or in combination) as foreground branches. Single-copy OGs were selected from our initial Orthofinder run, including orthologous with no missing data, and with missing data that exclude *V. infernalis*. Amino acid alignments generated with MAFFT were used to guide codon-based alignments, and columns with gaps were removed. We used the branch-site model A, with a model allowing *ω* > 1 in foreground branches. Sites under significant selection were identified based on the BEB method, retaining only those with BEB > 0.95. Statistical significance was evaluated by comparing the alternative model with a null model in which *ω* was fixed at 1 in the foreground branches, using a likelihood ratio test (ΔLRT = 2 × (ln*L*_1_ − ln*L*_0_)). ΔLRT values were compared to critical values of 2.71 (5% significance) and 5.41 (1% significance), assuming a null distribution modelled as a 50:50 mixture of a point mass at 0 and a *χ*^2^ distribution with 1 degree of freedom.

### Gene expression profiling

We explored the gene expression patterns across multiple tissues of *S. spirula* using bulk transcriptome data (Supplementary Table 9). Low-quality read pairs and illumina adaptor sequences were removed using fastp v0.23.2^98^. Cleaned reads were pseudo-aligned against the largest isoform per gene using Kallisto v0.46.1^121^ to estimate transcript abundance in transcripts per million across each tissue. Our analysis focused on the siphuncle, which is involved in the shell maintenance. Changes in gene expression were visualized using a heatmap of selected genes using Seaborn v0.11.2^140^ and Matplotlib v3.5.2^141^ in Python v3.8. Gene expression values were row-normalized using a *z*-score normalization.

### Reporting summary

Further information on research design is available in the Nature Portfolio Reporting Summary linked to this article.

## Supplementary information

Supplementary Information Supplementary Notes, Supplementary Fig. 1 and Supplementary Tables 1–7.

Reporting Summary

Supplementary Tables 8–10 Supplementary Tables 8–10.

## Data Availability

All the genomic and transcriptomic data generated from the newly sequenced species are found under the NCBI BioProject accession number PRJNA1332616. Genomes generated by the Aquatic Symbiosis Genomics Project of the Wellcome Sanger Institute are available under the NCBI Umbrella BioProject PRJEB64979. The resulting alignments and phylogenetic trees are available via GitHub at https://github.com/gushiro/decapodiforms_phylogenomics.

## Code availability

The relevant codes are available via GitHub at https://github.com/gushiro/decapodiforms_phylogenomics.
