# Supplementary material for: Rapid mid-Cretaceous diversification of squid and cuttlefish preceded radiation into coastal niches
Source: Nat Ecol Evol. 2026 Mar 30;10(4):662–76. doi: 10.1038/s41559-026-03009-1 (PMC13076209; doi:10.1038/s41559-026-03009-1)
Supplement: Supplementary file 1 — Supplementary Notes, Supplementary Fig. 1 and Supplementary Tables 1–7. [file 41559_2026_3009_MOESM1_ESM.pdf]

nature ecology & evolution

Supplementary information https://doi.org/10.1038/s41559-026-03009-1

Rapid mid-Cretaceous diversification of squid and cuttlefish preceded radiation into coastal niches

In the format provided by the authors and unedited

TABLE OF CONTENTS

SUPPLEMENTARY NOTES

Supplementary Note 1. Genome assembly, annotation, and comparative analysis
Supplementary Note 2. Phylogenomics and systematic implications in decapodiforms
2.1: Phylogenomics and alternative topologies
2.2: Naef's classic decapodiform suborders evaluated under our phylogeny
Supplementary Note 3. Macrosynteny evolution of decapodiforms
3.1: Summary of altered chromosome-scale synteny in Sepiolida and Idiosepiida
3.2: Search for syntenic synapomorphies.
Supplementary Note 4. Alternative calibration and divergence estimation
Supplementary Note 5. Biomineralization-related genes in decapodiforms
Supplementary Note 6. Genes found in the siphuncle of *S. spirula*.
Supplementary Note 7. Analysis of amino acid changes under positive selection.

SUPPLEMENTARY FIGURES

Supplementary Figure S1. Alternative divergence time estimates under four fossil calibration schemes.

SUPPLEMENTARY TABLES

Supplementary Table S1. Detailed classification corresponding to the category "Habitat" is shown in Figure 1.
Supplementary Table S2. Genome assembly statistics and data sources for species included in this study.
Supplementary Table S3. Genome annotation statistics for newly sequenced decapodiform species. The genome of *D. pealeii* is used as a reference.
Supplementary Table S4. Benchmarking Universal Single-Copy Orthologs (BUSCO) completeness scores and gene model structure summaries.
Supplementary Table S5. Convergence and mixing results for PhyloBayes runs under the CAT-GTR+Γ model.
Supplementary Table S6. Posterior predictive analyses under CAT-GTR+Γ, GTR, and LG models.
Supplementary Table S7. Phylogenetic markers identified to polarize chromosomal changes and infer ancestral states in the decapodiform stem.
Supplementary Table S8. Highly expressed genes in the siphuncle of the ram's horn *Spirula spirula*. (Excel sheet)
Supplementary Table S9. Orthogroups inferred to be under positive selection in selected branches. (Excel sheet)
Supplementary Table S10. RNA-seq read counts and tissue sources for the newly sequenced species in this study. (Excel sheet)

Supplementary Note 1. Genome assembly, annotation, and comparative analysis

We sequenced three additional decapodiform genomes to chromosome level, representing two previously unsampled lineages; *Idiosepius pygmaeus* from the order Idiosepiida and *Spirula spirula* from the order Spirulida, as well as an additional representative of the order Oegopsida, *Todarodes pacificus* (**Methods, Extended Data Figure S1A and S1B, Supplementary Table S2, S3)**.

Genome annotation was performed using BRAKER3 in combination with the Mikado pipeline to reduce fragmented gene models, followed by post-filtering steps to remove genes with significant similarity to transposable elements (TEs) and mono-exonic genes having no characterized functional domains (**Methods, Extended Data Figure S1A**). BUSCO analysis using the metazoan dataset indicates that this approach improved the completeness of gene models while reducing the proportion of fragmented and missing genes compared to previously published annotations (**Supplementary Table S4**).

To enable comparative analyses within Sepiida, we applied the same annotation pipeline to the draft genome of the pharaoh cuttlefish *Ac. pharaonis*[1]. For example, Song et al.[1] reported 89.7% completeness, 5.9% duplication, 10.2% fragmented, and 10.3% missing genes based on BUSCO analysis. In contrast, our annotation yielded 88.7% completeness, with only 0.6% duplication, 6.2% fragmented, and 5.1% missing genes (**Supplementary Table S4)**. The resulting gene models exhibit comparable completeness but markedly reduced duplication and fragmentation, providing a more robust reference for the Sepiida lineage.

We included six additional decapodiform genomes publicly available from the Aquatic Symbiosis Project by the Wellcome Sanger Institute, as well as the genome of the dwarf cuttlefish *As. bandense*[2]. Given that these genomes lacked existing gene annotations, we annotated these genomes using GALBA with gene models of closely related species, followed by post-filtering steps to remove genes with significant similarity to transposable elements (TEs) (**Methods, Extended Data Figure S1A**). Using this approach, we generated gene models for these newly available species and combined them with three annotated genomes from previous studies, resulting in a total of 14 decapodiform species for our analysis (**Supplementary Table S2)**.

Across our newly sequenced genomes, *S. spirula* has the largest genome (5.56 Gb) and the highest TE content (66.1%), with particularly high contributions from DNA transposons (15.8%) and LTR elements (11.32%). *T. pacificus* follows with a genome size of 2.74 Gb and a TE content of 58.9%, dominated by LINEs (13.65%) and DNA transposons (5.55%) (**Extended Data Figure S1C**). This is comparable to the TE landscape observed in *D. pealeii* (4.6 Gb), which has 46.9% TE content and, like *T. pacificus*, is enriched in non-LTR retrotransposons, particularly LINEs (14.45%) and followed by SINEs (6.07%)[3]. In contrast, *S. spirula* presents a more balanced repeat profile, in which LTR elements (11.3%) are nearly as abundant as LINEs (10.13%) and DNA transposon content (15.8%)(**Extended Data Figure S1C**). These results indicate lineage-specific differences in TE composition among decapodiform genomes, with

non-LTR retrotransposons predominating in some species, while others like *S. spirula* retain a more diverse repeat landscape.

Among the newly sequenced genomes, *I. pygmaeus* shows a high TE content and a distinct repeat landscape (**Extended Data Figure S1C**). It possesses the smallest genome (1.68 Gb) reported among coleoids yet displays the highest TE content (68.5%). LTR elements alone account for 18.6% of the genome, making them the dominant retroelement class, followed by LINEs (7.49%) and simple repeats (7.11%) (**Extended Data Figure S1C**). The high LTR content observed in this small and highly rearranged genome (**Figure 3**) might reflect a historical burst of retrotransposition followed by incomplete purging. This pattern could be explained by a low long-term effective population size, where genetic drift reduces the efficacy of purifying selection, allowing transposable elements to accumulate.

In addition, we found that *I. pygmaeus* has the lowest mean GC content across its chromosomes, which may reflect a reduced recombination rate and an overall decrease in genetic diversity (**Extended Data Figure S1D**). The pronounced bias toward AT alleles suggests that GC-biased gene conversion is weak in this lineage. This pattern indicates that in *I. pygmaeus*, natural selection is less effective, allowing genetic drift to play a dominant role in shaping the genome. The extensive chromosomal rearrangements observed in *I. pygmaeus* may thus reflect the long-term impact of drift under low effective population size. By contrast, *S. spirula* exhibits the highest mean GC content observed among all decapodiform lineages (**Extended Data Figure S1D**). This, together with the expansion of immune-related gene families (**Extended Data Figure S7B**) and its broad ecological distribution, supports the idea that *S. spirula* may experience higher levels of recombination and maintain a larger effective population size.

We were also able to identify the Z chromosome across all species using our synteny analysis (**Figure 3**). This chromosome consistently shows the lowest GC content among all chromosomes (**Extended Data Figure S1E**), which can be explained by its recombination landscape: in the ZZ/Z0 sex determination system of coleoid cephalopods[4,5], recombination on the Z chromosome occurs only in males, whereas autosomes recombine in both sexes.

Large chromosomes with low GC content and among the highest gene counts show conserved synteny and shared homology across species, including Scaffold2 in *S. spirula*, Chromosome 5 in *Ac. esculentum*, Dp15 in *D. pealeii*, Scaffold14 in *T. pacificus*, Scaffold20 in *E. berryi*, and Scaffold29 in *I. pygmaeus* (**Extended Data Figure S1F**). Low GC regions are more prone to structural instability, increasing the chance of gene duplication and deletion events. Consistent with this, these homologous chromosomes with low GC content across coleoids are enriched in expanded gene families, including Protocadherins and C2H2 zinc finger proteins; two gene families known to play key roles in neural development and gene regulation, and considered hallmarks of coleoid innovation.

Supplementary Note 2. Phylogenomics and systematic implications in decapodiforms

2.1: Phylogenomics and alternative topologies

We considered the effect of possible modeling artifacts due to cross-taxon and/or cross-site rate heterogeneity, long-branch attraction, and incomplete lineage sorting during rapid cladogenesis[6–8]. To test for rate heterogeneity we compared the CAT-GTR+Γ model to the simpler site-homogeneous models LG and GTR. Both produced identical topologies (**Figure 2B and 2C**) with Idiosepiida as the earliest diverging lineage, each with strong posterior support (pp = 1; **Figure 2B and 2C**). Nonetheless, posterior predictive analyses confirmed that the CAT-GTR+Γ model provides a significantly better fit to our dataset than LG and GTR, particularly in modeling both across-site and across-taxon compositional heterogeneity (**Supplementary Table S6**). This was the only model that showed no significant deviation between the mean and observed amino acid diversity per site (pp = 0.665), with the lowest absolute z-score (0.45) (**Supplementary Table S6**). In tests of compositional homogeneity across taxa, CAT-GTR+Γ also produced lower z-scores for all decapodiforms and outgroups, except for *I. pygmaeus*, which exhibited the highest level of compositional heterogeneity (**Extended Data Figure S3**). Similarly, the topologies from the less-well-fit LG and GTR models were also recovered with maximum likelihood analysis using the best-fit partitioning scheme and site-homogeneous model selection[9,10], indicating that site-homogeneous models behave similarly in Bayesian and likelihood approaches for various partitioning strategies. After removing the fast-evolving Idiosepiida and Sepiolida (**Extended Data Figure S4**), modeling incomplete lineage sorting within the coastal group recovered Myopsida and Sepiida as sister lineages, consistent with the relationship inferred under the CAT-GTR+Γ model (**Figure 2E**). To further test robustness, we added an additional octopodiform outgroup. Members of the suborder Cirrata would be the ideal complement to *V. infernalis* (**Figure 1**), given their phylogenetic position and sequence divergence[11], but genomic resources for this group are currently unavailable. To broaden our octopodiform outgroup sequences, we added *Argonauta argo* (suborder Incirrata)[12]. Nuclear-gene phylogenies place this species among the earliest-branching incirrates, but mitochondrial phylogenies suggest a placement within Incirrata, sister to Octopodidae[13]. Uncertainty about the detailed placement of *Ar. argo* within incirrates, however, does not affect our analysis.
The decapodiform phylogeny with our 361 high-quality orthogroups under CAT-GTR+Γ model with outgroups *Ar. argo*, *V. infernalis*, and *O. vulgaris* (**Extended Data Figure S2A**) have the same topology as that shown in **Figure 2**. Only the shortest internal branch decreased slightly in posterior support (pp = 0.99). These results support the use of CAT-GTR+Γ for resolving the relationships among decapodiform lineages.

To explore how datasets and model choice may have shaped the topologies reported in previous studies, we first analyzed our full set of 1,398 single-copy orthologous genes (OGs) present across all 14 decapodiform species and two octopodiform outgroups (**Methods**) using site-homogeneous partitioned maximum likelihood (ML) models. Under various site-

homogeneous models and partitioning schemes, we found three conflicting topologies, placing Idiosepiida, Sepiolida, or Sepiida as the earliest-diverging decapodiform lineage depending on the subset or method used.

Using the entire 1,398 OGs, ML analysis placed Idiosepiida as the earliest diverging lineage (bootstrap = 100), consistent with previous studies[14–16] (**Extended Data Figure S2B**). This analysis also placed Sepiida as sister to the Oegopsida + Spirulida clade (bootstrap = 100).

Gene concordance analysis across the 1,398 OGs, however, revealed substantial phylogenetic conflict, with 280 and 297 OGs supporting alternative topologies and 821 OGs showing discordance due to paraphyly. ML trees built from these 280 and 297 OGs subsets recovered Sepiolida or Sepiida as the earliest diverging lineage, respectively, both with strong support (**Supplementary Figures S2C and D**). Similarly, Sepiolida was also recovered as the earliest diverging lineage in a recent study by Chen et al.[17], which analyzed eight decapodiform genomes. However, their dataset included fewer representative taxa within major lineages and lacked both Spirulida and Idiosepiida, potentially contributing to the observed topology. Likewise, the placement of Sepiida as the earliest lineage (**Extended Data Figure S2C**) reflects alternative topologies proposed in earlier mitogenome and transcriptome studies[11,18,19].

Differences in modeling approaches likely also contributed to the contrasting topologies observed with our datasets and previous studies. Previous studies using ML-based finite mixture models with 16 categories[14–16] may be more prone to overfitting compared to the more flexible CAT-GTR + Γ model, which infers site categories from the data. Although Tanner et al.[11] also used CAT-GTR + Γ, their analysis relied on a smaller and less complete dataset compared to ours, with fewer genes (180 OGs vs. 361), shorter alignments (36,156 vs. 152,405 amino acid positions), and a higher proportion of missing data (26% vs. no-missing data), derived from transcriptomic sources rather than genome assemblies.

In addition to model choice, we reduce potential biases in orthology inference and sequence quality. Our pipeline applied stringent filtering to exclude genes with paralogs or long terminal branches. Our dataset relies almost entirely on protein sequences inferred from genomes, except for a predominantly non-neural arm-tip transcriptome of *V. infernalis* as one of two octopodiform outgroups. We note that neural transcriptomes in coleoids are subject to widespread A-to-I mRNA editing[3,20], which may alter the encoded protein and could lead to artifactual attraction between transcriptome-derived proteins due to shared edited sites and/or confound site-heterogeneous models.

Overall, the multiple topologies recovered in our analyses and previous studies suggest that taxon sampling, ortholog selection, dataset composition, and model choice play a critical role in shaping decapodiform phylogeny.

2.2: Naef's classic decapodiform suborders evaluated under our phylogeny

For much of the late 20th century, Naef's decapodiform classification[21] was widely accepted. Naef[21] classified living decapodiform cephalopods into two clades: Teuthoidea (grouping myopsids and oegopsids) and Sepioidea (including sepiids, spirulids, sepiolids, and idiosepiids). Further revision, such as the classifications of Clarke & Trueman (1988) and Sweeney (1998) generally followed Naef's classification and served as primary references prior to the World Register of Marine Species (WoRMS). With the advent of molecular phylogenetics, however, Naef's classification has been largely superseded [11,14–16,19,22]. Here we will evaluate Naef's groupings in our new phylogeny.

Naef[21] considered the clade Decapodiformes Leach 1817 to be an "order" and so designated Teuthoidea Naef 1916 and Sepiodea Naef 1916 as "suborders." Subclades within Teuthoidea and Sepioidea were therefore considered "families." Naef's "families," however, generally correspond to what are now recognized as Orders (Myopsida, Oegopsida, Sepiida, Sepiolida, Idiosepiida, and Spirulida), with Decapodiformes now considered to be a superorder. To avoid confusion, in the discussion of Naef's classification system under our new phylogeny, we will simply refer to Teuthoidea and Sepioidea as "clades."

For Teuthoidea, Naef[21] grouped myopsids with oegopsids based on (i) the reduction proostracum and the derived nature of the phragmocone in the gladius, (ii) the presence of a funnel incision of ventral mantle margin, (iii) wide gill axis connected to the mantle between the branchial lamellae, (iv) tricuspid rachidian radular teeth, and (v) their nektonic lifestyle.

In contrast, our new decapodiform phylogeny places myopsids and oegopsids in separate clades, with Myopsida grouped with Idiosepiida, Sepiolida, and Sepiida in a coastal and shallow-water clade and Oegopsida grouped with Spirulida in an open-ocean clade. Following our phylogeny (**Figure 2**), the gladius, mantle, and gill traits are convergent adaptations to the nektonic life shared by myopsids and oegopsids. Moreover, the rachidian radular tooth cusp number is variable within Oegopsida (e.g., it appears as unicuspid in some onychoteuthids[23], and the tricuspid form is also present in octopuses[24] and in non-coleoid extinct cephalopods[25], indicating that tricuspy is likely plesiomorphic or has evolved multiple times.

For Sepioidea, Naef[21] grouped sepiids and spirulids based on the homology between the sepiid cuttlebone and the spirulid phragmocone, and by the presence of intermediate fossils. Sepiolids and idiosepiids were further added to this group because of their similar gonadal attachment. However, while homologous, the sepiid cuttlebone differs structurally from the spirulid phragmocone in lacking a caecum[26]. Naef's clade would be consistent with a single origin of the caecum with secondary loss in sepiids.

In contrast, our phylogeny (**Figure 2**) places sepiids and spirulids in different higher-level clades and is consistent with independent re-elaboration of their internal shells from the ancestral decapodiform condition rather than a single shared-derived form.

Thus, our new phylogeny (**Figure 2**) suggests that several of the diagnostic characters described in Naef[21] as differentiating Teuthoidea from Sepioidea are better explained by convergence or by the retention of the characters from the ancestral decapodiform condition.

Among historical classifications of decapodiforms, our new phylogeny aligns more closely with d'Orbigny's (1841)[27] scheme, aside from his assignment of *Cranchia* and *Beloptera* to Myopsida, and Belemnitida to Oegopsida. We note that d'Orbigny used 'Myopsidès' (closed eyes) to group Sepiida, Sepiolida, and what is now called Myopsida (Loliginidae and Australiteuthidae; WoRMS, 2025), and 'Oigopsidès' (open eyes) for most other squids plus *S. spirula*. Consequently, his historical division broadly matches our newly described clades: Corneata (Sepiida, Myopsida, Idiosepiida, and Sepiolida) and Acorneata (Oegopsida and Spirulida).

Supplementary Note 3. Macrosynteny evolution of decapodiforms

3.1: Summary of altered chromosome-scale synteny in Sepiolida and Idiosepiida

Here, we summarize the changes observed in chromosome-scale synteny relative to the ancestral decapodiform karyotype. Since the karyotypes of our sequenced representatives of Oegopsida, Spirulida, Myopsida, Sepiida are in 1:1 correspondence, they all preserve the ancestral state (**Figure 3** and **Supplementary Note 3.2** below), and we can use any of these genomes to number the ancestral set. We use *D. pealeii* (DPE) to maintain consistency with prior work Albertin et al.[3].

With DPE chromosomes as a reference, we can polarize the direction of chromosomal change and identify derived rearrangement states specific to Sepiolida and Idiosepiida. For example, the chromosomal rearrangement in the sepiolid *Euprymna berryi* (EBE1), is a fusion that occurred between DPE24 and DPE40 via likely centric insertion (EBE1≡ DPE24↘DPE40). Here we follow the notation of Simakov et al.[28]. This fusion reduces the number of chromosomes to *N=45* on Sepiolida stem. The idiosepiid *Idiosepius pygmaeus* (IPY) does not exhibit this fusion, as DPE40 remains equivalent to IPY34 (DEP40 ≡ IPY34).

We also observe that IPY possesses the most rearranged chromosome set among decapodiforms sequenced to date, with only 19 chromosomes retaining the conserved syntenies observed among all lineages. The remaining chromosomes have undergone substantial rearrangements, primarily through fission and translocations, but also with cases of fusion. These extensive lineage-specific structural changes increased the total chromosome number of IPY to *N= 47*.

Signatures of translocations are the most extensively defined changes observed in IPY. For instance, reciprocal translocations are observed in the formation of IPY46 and IPY08, which arose from DPE13 and DPE34 (IPY46 ≡ DPE13a * DPE34a and IPY08 ≡ DPE13b * DPE34b). Additionally, we identified reciprocal translocation followed by mixing in one or both arms, as seen in IPY35 ≡ DPE21b * DPE35a and IPY19 ≡ DPE21* DPE35; and in IPY02, IPY07 ≡ DPE06 * DPE03. A non-reciprocal translocation was found in IPY06, where DPE25 incorporated fragments of DPE31 and DPE11 through fusion-with-mixing (IPY06 ≡ DPE25 + partial_DPE31 ⊗ partial_DPE11).

Fission events were also observed in IPY, involving the fragmentation of DPE02, DPE11 and DPE18, each splitting into two or three IPY chromosomes (DPE02 → IPY21 and IPY05; DPE11 → IPY06, IPY21, and IPY05; DPE17 → IPY17, IPY37, and IPY24). Similarly, the fission of DPE08 and DPE30 generated IPY27, IPY27 ≡ partial_DPE08 + partial_DPE30.

We also detected fusion events, although they are less frequent than fission and translocation. These include Robertsonian translocation, such as IPY11 ≡ DPE12 ● DPE27, as well as other fusion events followed by mixing, such as IPY09, which resulted from the from DPE30 and part of DPE08 (IPY09 ≡ DPE30 ⊗ partial_DPE08). Centric insertions were observed in the formation of IPY15, IPY15 ≡ DPE14 ↘ partial_DPE16.

Finally, a minor rearrangement included IPY12, which consisted mostly of DPE16 (IPY12 ≡ most_DPE16).

Overall, our results demonstrate that while chromosomal synteny remains largely conserved across most decapodiform lineages, substantial structural rearrangements can accumulate in specific lineages, particularly those experiencing unique evolutionary pressures.

3.2: Search for syntenic synapomorphies.

Given that *I. pygmaeus* (IPY) occupies a derived position in our phylogeny (**Figure 2**), we examined patterns of macrosynteny to further support this placement and clarify the direction of chromosomal changes. By comparing conserved synteny among IPY, DPE, *Octopus vulgaris* (OCT), and *Nautilus pompilius* (NAU), we assessed the polarity of chromosomal changes using 5,801 single-copy orthologs shared across all four species (**Methods**), and evaluated whether synteny patterns reflect the same phylogenetic signal.

Four OCT chromosomes; OCT12, OCT15, OCT16, and OCT20, displayed conserved synteny with both DPE and IPY. Among them, OCT15 showed a 1:1 correspondence with IPY13 and included most of DPE04, while OCT16 corresponded to DPE43 and IPY43 (**Extended Data Figure S5B**, **Supplementary Table S7**). These nearly perfect stable relationships across all three species likely reflect deeply conserved chromosomal segments and are thus uninformative for polarizing rearrangements. In contrast, OCT12 and OCT20 showed clear 1:1 correspondence with DPE03 and DPE11, respectively, but fragmented synteny with IPY. Specifically, OCT12 mapped to IPY2 and IPY7 (1:2), and OCT20 to IPY5, IPY6, and IPY21 (1:3), with orthologs distributed roughly evenly across the IPY chromosomes (**Extended Data Figure S5C**, **Supplementary Table S7**). These patterns support DPE as retaining the ancestral decapodiform chromosomal state, while the IPY configuration reflects secondary rearrangements consistent with its derived position.

We also compared OCT and NAU to evaluate whether any synteny signal could be recovered at the base of the coleoid lineage. Several OCT chromosomes appear to have arisen through fusion-with-mixing events that involve multiple NAU chromosomes, and no clear 1:1 correspondences were observed (**Extended Data Figure S5D**, **Supplementary Table S7**). This likely reflects extensive rearrangements early in the coleoid stem lineage, limiting the utility of NAU as an outgroup for polarizing more recent chromosomal changes.

While only a few OCT chromosomes, specifically OCT12 and OCT20, show clear synteny with DPE03 and DPE11, respectively, they serve as reliable phylogenetic markers. The patterns of rearrangement observed in IPY are consistent with its derived position. Given the 1:1 chromosomal correspondence among Myopsida (*D. pealeii*), Oegopsida (*T. pacificus*), Spirulida (*S. spirula*), and Sepiida (*Ac. esculentum*), the conserved arrangements in any of these lineages can be used to support interpretations of the ancestral decapodiform karyotype.

Supplementary Note 4. Alternative calibration and divergence estimation

By using the fossil lower beaks identified as from oegopsids and myopsids by Ikegami et al.[29], and a root age of 240–245 Mya, we obtained a crown decapodiform origin point estimate in the mid-Cretaceous at 101 Mya (95% CI: 122.6–99) (**Figure 4, Supplementary Fig. S1A**). In the resulting chronogram, the estimate for most recent common ancestor (MRCA) of Oegopsida and Spirulida almost completely overlaps this range (95% CI: 122.0–99 Mya) with a point estimate of 99 Mya at the oegopsid constraint from Ikegami et al.[29] An early origin for the spirulid lineage is consistent with the earliest known stem spirulid fossil of Groenlandibelidae at ~72 Mya[30]. Thus, adding a time constraint for the spirulid lineage to our analysis based on this fossil would not change our results, as we confirmed directly.

We further consider the impact of fossil calibrations on divergence time estimates. The simplest calibration only constrains the coleoid root (common ancestor of squid, cuttlefish, and octopuses) to occur between 240 and 245 Mya, based on the fossil record of *Germanoteuthis donai* and *Breviconoteuthis breviconus,* true coleoids (also known as neocoleoids or proostracum-bearing coleoids) from the Triassic Ladinian and Asinian, respectively[31–33]. Using only this coleoid root calibration, a molecular clock calculation finds a more recent origin for Decapodiformes than the full analysis shown in **Figure 4**, with a point estimate of ~67 Mya (95% CI: 84.9–54.2 Mya) (**Supplementary Fig. S2B)**. The estimated divergence of Myopsida (i.e., the *Doryteuthis-Sepioteuthis* split in our data) ~31 Mya (95% CI: 40.6–22.7) and the split of Spirulida from Oegopsida at ~55 Mya (95% CI: 68.3–43.8 Mya) are substantially younger than fossil evidence suggests (~100–94 Mya for loliginids (Myopsida)[29] and ~72 Mya for stem spirulids[30], respectively). These more recent age ranges suggest that molecular evolution within decapodiforms was slower than along decapodiform stem, and that lineage-specific rate variation within decapodiforms may bias molecular clock estimates when fossil constraints are not applied (**Supplementary Fig. S2B**).

Ikegami et al.'s[29] recent "digital fossil mining" of fossilized squid lower beaks from carbonate concretions of Cretaceous shelf deposits in Japan[29] revealed that (1) beaks that could clearly be assigned to loliginids (the family within Myopsida to which both *Doryteuthis* and *Sepioteuthis* belong) and (2) four new lineages of oegopsid-like beaks that could not be assigned to any living oegopsid families. The oldest of these were in the Cenomanian (100.5-93.9 Mya).

Since the myopsid fossils were most confidently assigned, we added a constraint to our chronogram that the most recent common ancestor (MRCA) of Myopsida, Idiosepiida, and Sepiolida must be older than the midpoint of the Cenomanian, since putative myopsids can only occur after the cladogenic event that produced the myopsid lineage (**Figure 2**). With this calibration (and the coleoid root constraint), the crown decapodiform group estimate is pushed back to a midpoint estimate of 101 Mya, with 95% CI (119.4–99 Mya) that spans the boundary between the Early and Late Cretaceous (**Supplementary Fig. S2C)**. The more recent side of the CI at the fossil calibration is explained by the slow rate of evolution in decapodiforms, which led to a coleoid-constraint only estimate that was much younger. With only this myopsid calibration

added, the estimated age of the MCRA of Oegopsida–Spirulida is pushed back to ~76.3 (95% CI: 89.1–67.7 Mya), and now consistent with the ~72 Mya fossil spirulid Groenlandibelidae at ~72 Mya[30] without directly constraining the Spirulida node (**Supplementary Fig. S2C**). If we further constrain the chronogram analysis by requiring that the Spirulida-Oegopsida split occurs before than the oldest known spirulid fossil (~72 Mya), the point estimate for the crown age of decapodiforms remains 101 Mya (95% CI: 119.4–99 Mya), and other estimates are only slightly modified (**Supplementary Figure S1D).** Most notably, with the addition of the spirulid constraint the 95% CI for the Spirulida-Oegopsida split is now bounded by 72 Mya (and this is also the point estimate for this node). Thus, as far as early decapodiform branchings are concerned, the spirulid constraint does not add new information beyond that provided by the myopsid fossils.

Finally, we considered the Late Cretaceous fossil beaks that Ikegami et al.[29] attributed to stem oegopsids. Taking their interpretation at face value, their data constrain the origin of the oegopsid lineage (the Oegopsida-Spirulida split in our phylogeny) as being older than their oldest such fossil (Cenomanian, 100.5-93.9 My). Implementing this as a further constraint that the Oegopsida-Spirulida node be older than 99 Mya produced the chronogram shown in main **Figure 4** and repeated as **Supplementary Figure S1A** with confidence intervals shown. Adding this constraint to the coleoid node and myopsid and spirulid lineage constraints, has limited impact on almost all timings relative to the chronogram with only the myopsid lineage constraint. The only notable difference is the shift of the oegopsid-spirulid node to be older and closer to the decapodiform MRCA, consistent with the short branch lengths found in our molecular phylogeny (**Figure 2**).

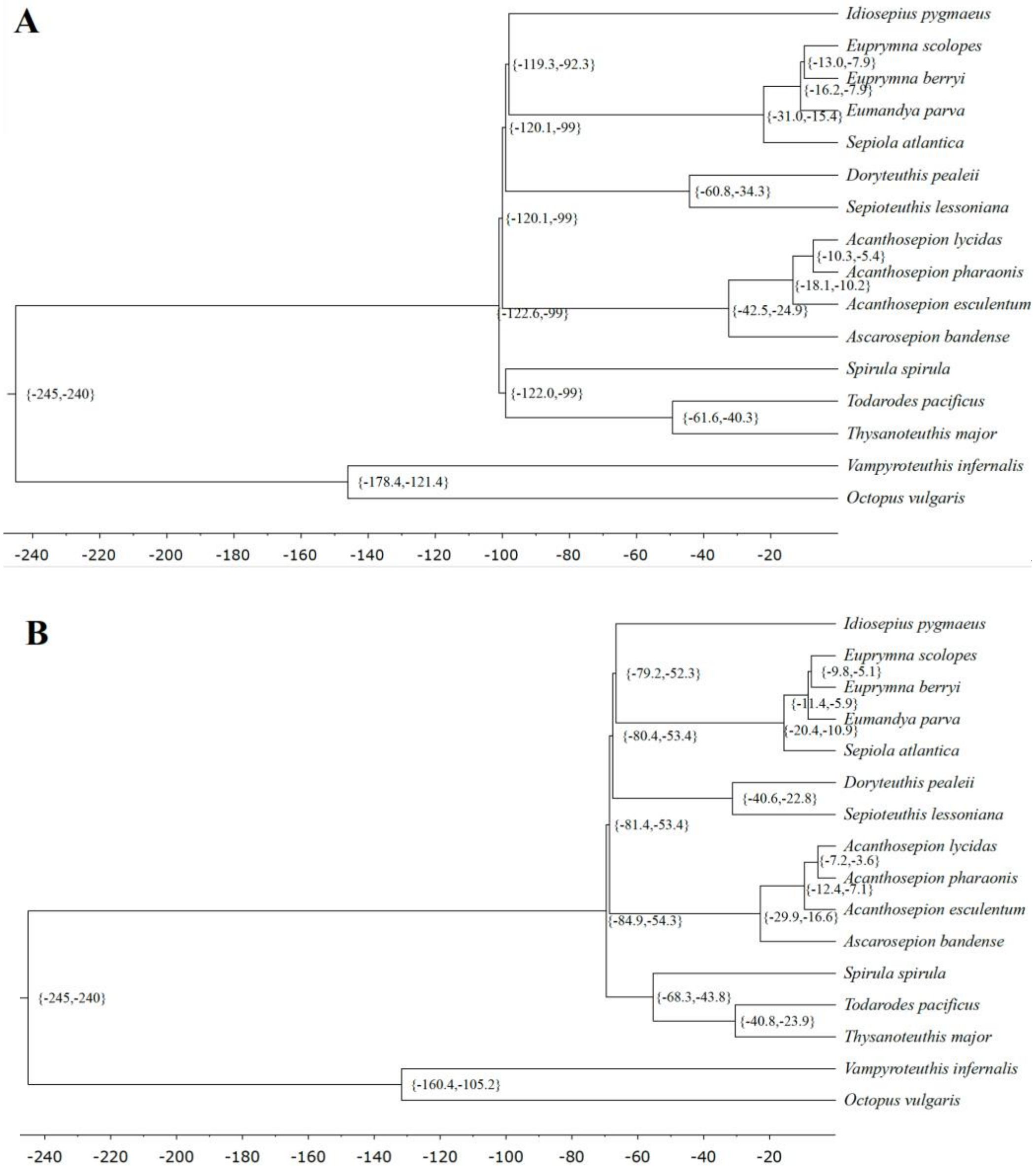
A
Idiosepius pygmaeus
Euprymna scolopes
{-13.0,-7.9}
Euprymna berryi
{-16.2,-7.9}
Eumandya parva
{-31.0,-15.4}
Sepiola atlantica
Doryteuthis pealeii
{-60.8,-34.3}
Sepioteuthis lessoniana
Acanthosepion lycidas
{-10.3,-5.4}
Acanthosepion pharaonis
{-18.1,-10.2}
Acanthosepion esculentum
{-42.5,-24.9}
Ascarosepion bandense
Spirula spirula
Todarodes pacificus
{-61.6,-40.3}
Thysanoteuthis major
Vampyroteuthis infernalis
{-178.4,-121.4}
Octopus vulgaris
{-119.3,-92.3}
{-120.1,-99}
{-120.1,-99}
{-122.6,-99}
{-122.0,-99}
{-245,-240}
-240 -220 -200 -180 -160 -140 -120 -100 -80 -60 -40 -20
B
Idiosepius pygmaeus
Euprymna scolopes
{-9.8,-5.1}
Euprymna berryi
{-11.4,-5.9}
Eumandya parva
{-20.4,-10.9}
Sepiola atlantica
Doryteuthis pealeii
{-40.6,-22.8}
Sepioteuthis lessoniana
Acanthosepion lycidas
{-7.2,-3.6}
Acanthosepion pharaonis
{-12.4,-7.1}
Acanthosepion esculentum
{-29.9,-16.6}
Ascarosepion bandense
Spirula spirula
{-68.3,-43.8}
Todarodes pacificus
{-40.8,-23.9}
Thysanoteuthis major
Vampyroteuthis infernalis
{-160.4,-105.2}
Octopus vulgaris
{-79.2,-52.3}
{-80.4,-53.4}
{-81.4,-53.4}
{-84.9,-54.3}
{-245,-240}
-240 -220 -200 -180 -160 -140 -120 -100 -80 -60 -40 -20

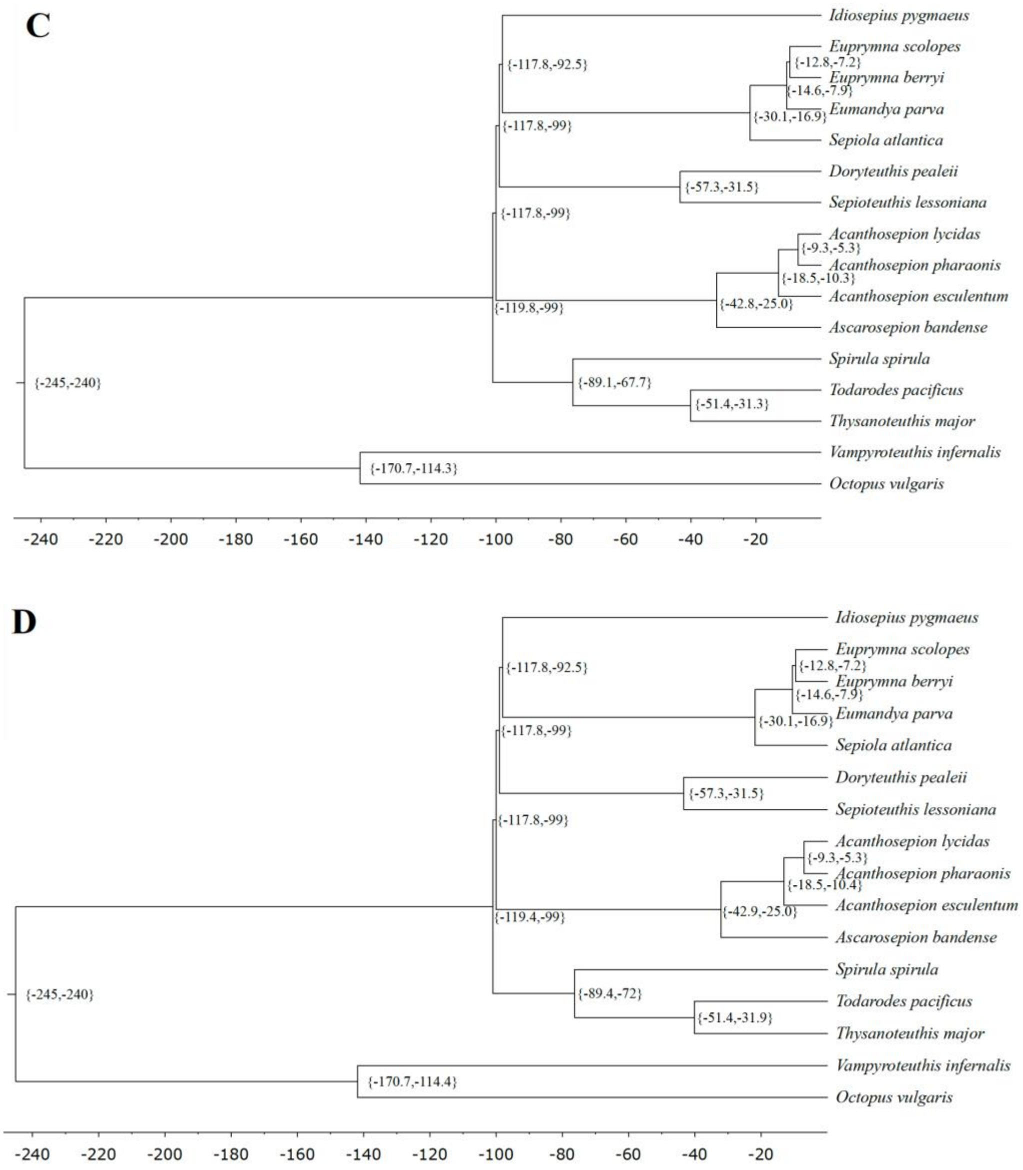

Supplementary Figure S1. Alternative divergence time estimates under four fossil calibration schemes. (A) Full calibration scheme using a prior at the coleoid root, plus lower beak fossils assigned to myopsids and oegopsids (same as main Figure 4), (B) with only prior at the coleoid root and no other fossil calibration, (C) with prior at the root plus lower beak fossil assigned to myopsids, and (D) with prior at the root plus lower beak fossil from myopsids and a shell fossil from stem Spirulida

Supplementary Note 5. Biomineralization-related genes in decapodiforms

PIF and its homologs, *LAMG3* and the blue mussel shell protein (*BMSP*), are core genes required for shell biomineralization in mollusks[34]. These proteins are typically characterized by a signal peptide and a combination of conserved functional domains. PIF contains a single von Willebrand factor A (vWFA) domain followed by one to three chitin-binding (CB) domains. BMSP, in contrast, is characterized by a tandem expansion of both vWFA and CB domains. LAMG3 lacks the vWFA domain but includes CB domains and an additional concanavalin A-like lectin/glucanase (LG) domain[35]. Although proteins containing the full complement of vWFA, CB, and LG domains have been referred to as the ancestral form of molluscan PIF ("Ancestral PIF")[34,36], recent studies have shown that this gene is present not only in mollusks and brachiopods, but also in other lophotrochozoans and chordates[37]. In our analysis, we identified this gene and, for simplicity, refer to it as Ancestral PIF (*PIF-A*) to distinguish it from the canonical molluscan *PIF*.

In cephalopods, *PIF* and *LAMG3* genes have been identified in the genomes of *Nautilus* as well as in octopuses, including those that lack shells and those that produce a shell-like egg case at adulthood, such as in *Argonauta*[12]. In our analysis, we identified one gene family comprising the *PIF* homologs (*PIF97* and *A. PIF*) and a separate gene family corresponding to *LAMG3*. In some species, however, only partial gene models for these families were initially recovered. We interpret these partial sequences as artifacts of incomplete or fragmented gene annotations. To improve detection and annotation, we re-searched the genomes using Miniprot 0.13-r248[38], employing closely related full-length protein sequences as queries and verifying that the hits mapped to the same genomic loci as the initially recovered partial gene models. Miniprot was run with the following parameters: --outc=0.5, --outs=0.95 to 0.90, --outn=1, -M 0, -K 5. This approach allowed us to recover homologs with a more complete set of expected functional domains. For example, *PIF97* from *S. spirula* was initially annotated without a signal peptide domain, but our Miniprot search recovered the full-length protein at the same genomic loci. The presence of this gene was further supported by RNA-seq evidence, which showed that *PIF97* was uniquely expressed in the siphuncle.

We found that *PIF97* or *PIF-A* has been lost in the common ancestor of Sepiolida and Idiosepiida, and that most decapodiforms retain only one of the two PIF homologs. An exception is *St. lessoniana*, which possesses both versions of the gene (**Figure 5B**). In addition, we were unable to detect *LAMG3* in *S. spirula* (**Figure 5B**), suggesting an evolutionary trajectory for its chambered shell that does not rely on *LAMG3* as in other decapodiforms. To explore this further, we characterized gene expression in the siphuncle and found high expression of PIF97 along with other key genes involved in molluscan shell biomineralization (**Supplementary Table S8**). These results support the idea that the siphuncle plays an active role in shell maintenance, beyond its well-known function in buoyancy control. Finally, we newly identified transcription factors and signaling molecules that may contribute to the unique regulatory program underlying shell formation in *S. spirula*.

Supplementary Note 6. Genes found in the siphuncle of *S. spirula*.

In addition to the biomineralization genes discussed in **Supplementary Note 5**, transcriptome analysis of the siphuncle revealed a diverse set of genes involved in extracellular matrix organization, tissue remodeling, regulatory signaling, and metabolic processes. These included the Procollagen lysine hydroxylase (*PLOD*) enzyme involved in collagen modification and indicating the role of collagen stabilization in the shell matrix; and matrix metalloproteinases (*MMP-21*, *MMP-3*) likely involved in extracellular matrix turnover.

We also detected transcription factors such as *BarH-like 1*, *Lhx1*, and *HLX1*, along with the signaling molecule Sonic Hedgehog (*SHH*), suggesting that regulatory pathways involved in cell differentiation and tissue patterning may have been co-opted for siphuncle function, providing molecular evidence for a unique regulatory program underlying shell formation in *S. spirula*.

In addition, we also found genes previously reported in the *S. spirula* shell proteome[39], including chitin deacetylases (*CDA1*, *CDA8*), collagens (*COL12A1*), and serine proteases (*TMPRSS3*, *PRSS30*, *APEH*). Together, these genes reinforce the siphuncle's role in both the structural assembly and dynamic remodeling of the shell matrix.

Several genes previously identified in the *S. spirula* shell proteome by Outdot et al.,[39], including chitin deacetylases (*CDA1*, *CDA8*), collagens (*COL12A1*), and serine proteases (*TMPRSS3*, *PRSS30*, *APEH*), were also expressed in the siphuncle. We also identified several previously unreported matrix metalloproteinases (*MMP-21*, *MMP-3*)(**Figure 5C**). Together, these genes reinforce the siphuncle's role in both the structural assembly and dynamic remodeling of the shell matrix.

We also detected transcription factors such as *BarH-like 1*, *Lhx1*, and *HLX1*, along with the signaling molecule Sonic Hedgehog (*SHH*) (**Figure 5C**), suggesting that regulatory pathways involved in cell differentiation and tissue patterning may have been co-opted for siphuncle function, providing molecular evidence for a unique regulatory program underlying shell formation in *S. spirula*.

Furthermore, we identified the Carbamoyl Phosphate Synthetase *CPS*, an ammonium metabolizing enzyme that may play a role in nitrogenous waste processing or pH homeostasis within the siphuncle (**Figure 5C**). Although *S. spirula* primarily relies on gas exchange for buoyancy, the presence of *CPS* suggests that it might also incorporate ammonium processing, either as a supplementary buoyancy mechanism or as part of broader metabolic regulation in the siphuncle. We also identified Aquaporin-9 *AQP9*, a gene that facilitates the transport of small solutes, including water, indicating a role in regulating fluid balance within the siphuncle, contributing to buoyancy control (**Figure 5C**). Overall, our findings reveal that the siphuncle plays a broader physiological role beyond gas exchange, highlighting its unique regulatory function in shell maintenance, matrix remodeling, and chamber fluid regulation, which distinguishes the *S spirula* shell from those of other extant decapodiforms.

Supplementary Note 7. Analysis of amino acid changes under positive selection.

RALBP (Retinal binding protein): Positive selection in RALBP in Idiosepiida and Sepiolida was detected at two residues: C40T (BEB = 0.98) located within the CRAL-TRIO lipid-binding domain where retinal binds, and N225G, (BEB = 0.969) in the GOLD domain involved in protein trafficking [40] (**Extended Data Figure S6A**). The loss of cysteine (C) in favor of a less rigid threonine (T) may have enhanced the release and uptake of retinal in conditions with greater light cycling exposure. On the other hand, the replacement of asparagine (N) with the smallest amino acid glycine (G) may have increased RALBP flexibility and facilitated more rapid intracellular trafficking.

ATP5F1C (ATP synthase subunit gamma): Positive selection in ATP5F1C in Oegopsida was detected at a single residue, V64T (BEB = 0.992) (**Extended Data Figure S6A**). This substitution replaces a hydrophobic valine with a polar threonine, potentially disrupting tight hydrophobic packing and increasing local flexibility. Such flexibility could facilitate conformational shifts during the mechanical rotation of the gamma subunit, potentially improving the coupling efficiency of ATP synthesis under high proton flux. While the precise functional consequences of the observed substitutions in ATP5F1C remain uncertain, we speculate that they may reflect lineage-specific adaptations of the Ommastrephidae (represented in our dataset by *Todarodes pacificus*) and Thysanoteuthidae (represented in our dataset by *Thysanoteuthis major*) to the elevated metabolic demands associated with fast, sustained swimming in pelagic environments.

IDH3B (**Isocitrate dehydrogenase subunit beta**). Positive selection in IDH3B in Oegopsida was detected at two sites: S98P/Q (BEB = 0.984) and D196K (BEB = 0.984). At position 98, *Th. major* possesses glutamine (Q), while *T. pacificus* presents proline (P), both derived from an ancestral serine (S) (**Extended Data Figure S6A**). These substitutions may represent a trajectory of increasing structural specialization, with glutamine enhancing hydrogen bonding and contributing to moderate stabilization, while proline introduces conformational rigidity by restricting backbone flexibility. Such changes may support greater catalytic stability or performance under elevated metabolic demands. The substitution at site 196, from aspartic acid (D) to a basic lysine (K), may also have functional implications. As with our discussion of selected sites in ATP5F1C above, we speculate that modifications to IDH3B in Oegopsida are related to the elevated metabolic demands associated with fast, sustained swimming in pelagic environments, but note that they may instead reflect parallel lineage-specific adaptations of the Ommastrephidae (represented in our dataset by *Todarodes pacificus*) and Thysanoteuthidae (represented in our dataset by *Thysanoteuthis major*).

SUPPLEMENTARY TABLES

Supplementary Table S1. Detailed classification corresponding to the category “Habitat” is shown in **Figure 1**.

**Orders**	Position relative to the sea floor	Depth in the water column	Depth of the Sea floor	Summarized Habitat Category
Sepiolida	Pelagic or benthic (depending on the subfamily)	Mesopelagic (subfamily Heteroteuthinae)	Benthic species: from very shallow to upper bathyal; pelagic species: all depths below shelf	Coastal/Neritic/Pelagic
Idiosepiida	Demersal	-	Very shallow (i.e., upper infralittoral)	Upper infralitoral / Shallow coastal
Sepiida	Demersal	-	To about 600 m	Coastal/Neritic
Myopsida	Demersal	-	To about 500 m	Coastal/Neritic
Spirulida	Pelagic	Mesopelagic	Upper slope	Open-ocean pelagic (mesopelagic)
Oegopsida	Pelagic or Demersal	Epi- and abyssopelagic	All	Open-ocean pelagic (epi- and abyssopelagic)
Bathyteuthida	Pelagic	Meso- to bathypelagic	All	Open-ocean pelagic (meso- and bathypelagic)
Vampyromorpha	Pelagic	Mesopelagic	All depths below shelf	Open-ocean pelagic (mesopelagic)
Cirrata	Demersal		All depths below shelf	Deep-sea demersal
Incirrata	Benthic (very few highly adapted pelagic species)	Mesopelagic (family Amphitretidae); Epi-bathypelagic (family Argonautidae depending on genus)	Benthic: all depths to lower bathyal; pelagic: all depths.	Coastal/deep-sea demersal

The summarized habitat classification collapses a three-dimensional distribution (position relative to the sea floor, depth in the water column, and proximity to shore) into a single descriptor for simplicity. This classification is guided by the following ecological categories:

- Depth gradient: Upper infralittoral → Neritic → Epipelagic → Mesopelagic → Bathypelagic → Demersal → Abyssal
- Proximity to shore: Coastal (intertidal, shallow shelf) vs. open ocean (oceanic, deep pelagic)

Supplementary Table S2. Genome assembly statistics and data sources for species included in this study.

Species	Lineage	Total length (GB)	N50 (MB)	Est. chrom #	Source
Idiosepius pygmaeus	Idiosepiida	1.63	35.48	47**(*)**	This study
Todarodes pacificus	Oegopsida	2.74	62.62	46**(*)**	This study
Spirula spirula	Spirulida	4.55	96	46**(*)**	This study
Thysanoteuthis major	Oegopsida	2.74	60.64	46	Wellcome Sanger Institute
Acanthosepion pharaonis	Sepiida	4.79	1.93	-	Song et al., 2021[1]
Acanthosepion lycidas	Sepiida	5.16	110.89	47	Wellcome Sanger Institute
Acanthosepion esculentum	Sepiida	5.1	111.5	46	Wellcome Sanger Institute
Ascarosepion bandense	Sepiida			-	Lorig-Roach et al., 2024 [2]
Sepioteuthis lessoniana	Myopsida	5.06	96.87	44	Sanchez et al., 2025[41]
Doryteuthis pealeii	Myopsida	4.9	107.4	46	Albertin et al., 2022[3]
Sepiola atlantica	Sepiolida	5.59	122.44	41	Wellcome Sanger Institute
Euprymna berryi	Sepiolida	5.92	113.96	45**(*)**	Gavriouchikina et al., 2025[42]
Euprymna scolopes	Sepiolida	5.12	120.3	45 (**)	Rogers et al., 2024[43]
Eumandya parva	Sepiolida	4.4	90.9	47	Wellcome Sanger Institute
Octopus vulgaris	Octopoda	2.8	118.9	30	Destanovi et al., 2023[44]
Vampyroteuthis infernalis (+)	Vampyromorpha	-	-	-	[SOURCE OF RNASEQ]

(*) Estimate values of chromosome number based on macrosynteny plots (**Figure 3**).
(**) Estimated based on Albertin et al., 2022[3]
(+) In the absence of an available *V. infernalis* genome sequence, we assembled a transcriptome from publicly available RNA-seq data.

Supplementary Table S3. Genome annotation statistics for newly sequenced decapodiform species. The genome of *D. pealeii* is used as a reference.

**Metric**	***Idiosepius pygmaeus***	***Spirula spirula***	***Todarodes pacificus***	***Doryteuthis pealeii***
Genome Size (bp)	1,631,521,633	4,548,522,109	2,741,097,045	4,598,076,745
Number of Genes	19,610	51,200	23,072	24,931
Total Gene Length (bp)	711,944,626	1,758,914,159	1,051,300,051	806,284,576
Total mRNA Length (bp)	711,944,626	1,758,914,159	1,051,300,051	806,284,576
Total CDS Length (bp)	27,362,541	55,036,495	28,673,067	26,765,529
Total Intron Length per CDS (bp)	560,950,817	1,520,645,785	845,302,0433	629,298,464
Intergenic Total Length (bp)	919,577,007	2,789,607,950	1,689,796,994	3,791,792,169
Mean Gene Length (bp)	36,305	34,354	45,550	32,340
Mean mRNA Length (bp)	36,305	34,354	45,550	32,340
Mean CDS Length (bp)	1,396	1,074	1,683	1,073
Mean Intron in CDS Length (bp)	4,860	13,396	6,468	6,122

Supplementary Table S4. Benchmarking Universal Single-Copy Orthologs (BUSCO) completeness scores and gene model structure summaries.

Species	Lineage	Complete (%)	Single-copy (%)	Duplicated (%)	Fragmented (%)	Missing (%)	mono: multi exonic ratio
Idiosepius pygmaeus (#)	Idiosepida	95.4	94.8	0.6	0.4	4.2	0.3
Todarodes pacificus (#)	Oegopsida	96.9	95.5	1.4	1.3	1.8	0.27
Spirula spirula (#)	Spirulida	89.1	85.1	4.0	1.9	9.0	1.21
Thysanoteuthis major (*)	Oegopsida	92.1	86.8	5.3	3	4.9	0.28
Acanthosepion pharaonis	Sepiida	88.7	88.1	0.6	6.2	5.1	0.56
Acanthosepion lycidas (*)	Sepiida	88.4	85	3.4	4.6	7	1.37
Acanthosepion esculenta (*)	Sepiida	85.6	84.9	0.7	5.1	9.3	1.48
Ascarosepion bandense (*)	Sepiida	83.8	82.5	1.3	4.8	11.4	1.16
Sepioteuthis lessoniana (*)	Myopsida	86.8	81.3	5.5	4.3	8.9	0.66
Doryteuthis pealeii	Myopsida	81.5	78.8	2.7	5.2	13.3	1.03
Sepiola atlantica (*)	Sepiolida	84.2	81.8	2.4	6.8	9	0.7
Euprymna berryi	Sepiolida	91.8	90.9	0.9	5.6	2.6	1.15
Euprymna scolopes	Sepiolida	83.7	80.6	3.1	10.3	6	0.31
Eumandya parva (*)	Sepiolida	81.9	79.7	2.2	9.6	8.5	1.72
Octopus vulgaris	Octopoda	91.2	90.7	0.5	3.5	5.3	0.13
Vampyroteuthis infernalis (+)	Octopoda	74.6	73.9	0.7	3.1	22.3	-

(#) gene models from species sequenced in this study
(*) gene models were generated using proteome mapping from the set of a closely related species
(+) derived from transcriptome assembly

Supplementary Table S5. Convergence and mixing results for PhyloBayes runs under the CAT-GTR+Γ model.

	6,600 MCMC	
**name**	**effsize**	**rel_diff**
loglik	118	0.179055
length	947	0.0816322
alpha	99	0.281606
Nmode	528	0.0324572
statent	79	0.164891
statalpha	249	0.0822257
rrent	1281	0.198758
rrmean	5606	0.0221644

Both the maxdiff and meandiff were 0

Supplementary Table S6. Posterior predictive analyses under CAT-GTR+Γ, GTR, and LG models.

Model	Observed Diversity (obs div)	Mean Diversity (mean div)	Standard Deviation (±)	Z-score	Posterior Probability (pp)
CAT-GTR+Γ	2.00394	2.00265	0.00285209	-0.451935	0.665111
GTR	2.00394	2.09344	0.00309362	28.9323	0
LG	2.00394	2.09301	0.00320404	27.8006	0

Supplementary Table S7. Phylogenetic markers identified to polarize chromosomal changes and infer ancestral states in the decapodiform stem. NAU: *Nautilus pompilius*; OCT: *O. vulgaris*; DPE: *D. pealeii*; IPY: *I. pygmaeus*.

Nautilus Chromosomes	OCT Chromosome	DPE Chromosome	IPY Chromosome	Fusion-Mixing Events	Notes
NAU1, NAU5	OCT12	DPE03	IPY7, IPY2	Fusion-mixing with clear 1:1	Strong marker supporting OCT-DPE relationship.
NAU12, NAU16, NAU25	OCT20	DPE11	IPY21, IPY5	Fusion-mixing with clear 1:1	Strong marker supporting OCT-DPE relationship.
NAU1, NAU6, NAU14	OCT16	DPE43	IPY43	Shared fusions, inconclusive	Unreliable as a marker. Equal correspondence in the three species.
NAU11, NAU23	OCT15	DPE04 (mostly)	IPY3	(almost) shared fusions, inconclusive	Mostly DPE04; only a few genes make it not 1:1. Unreliable as a marker.

Supplementary Table S8. Highly expressed genes in the siphuncle of the ram's horn *Spirula spirula*. (Excel sheet)

Supplementary Table S9. Orthogroups inferred to be under positive selection in selected branches. (Excel sheet)

Supplementary Table S10. RNA-seq read counts and tissue sources for the newly sequenced species in this study. (Excel sheet)

REFERENCES

1. Song, W. *et al.* Pharaoh cuttlefish, *Sepia pharaonis*, genome reveals unique reflectin camouflage gene set. *Front. Mar. Sci.* **8**, (2021).
2. Lorig-Roach, R. *et al.* Phased nanopore assembly with Shasta and modular graph phasing with GFAse. *Genome Res.* **34**, 454–468 (2024).
3. Albertin, C. B. *et al.* Genome and transcriptome mechanisms driving cephalopod evolution. *Nat. Commun.* **13**, 2427 (2022).
4. Coffing, G. C. *et al.* Cephalopod sex determination and its ancient evolutionary origin. *Curr. Biol.* **35**, 931–939.e4 (2025).
5. Torrado, H. *et al. Nautilus* sex determination is unique among cephalopods. *Curr. Biol.* **35**, 4561–4569.e3 (2025).
6. Mirarab, S. *et al.* ASTRAL: genome-scale coalescent-based species tree estimation. *Bioinformatics* **30**, i541–8 (2014).
7. Mirarab, S. & Warnow, T. ASTRAL-II: coalescent-based species tree estimation with many hundreds of taxa and thousands of genes. *Bioinformatics* **31**, i44–52 (2015).
8. Zhang, C. & Mirarab, S. Weighting by Gene Tree Uncertainty Improves Accuracy of Quartet-based Species Trees. *Mol Biol Evol* **39**, (2022).
9. Kalyaanamoorthy, S., Minh, B. Q., Wong, T. K. F., von Haeseler, A. & Jermiin, L. S. ModelFinder: fast model selection for accurate phylogenetic estimates. *Nat. Methods* **14**, 587–589 (2017).
10. Minh, B. Q. *et al.* IQ-TREE 2: New Models and Efficient Methods for Phylogenetic Inference in the Genomic Era. *Mol. Biol. Evol.* **37**, 1530–1534 (2020).
11. Tanner, A. R. *et al.* Molecular clocks indicate turnover and diversification of modern

coleoid cephalopods during the Mesozoic Marine Revolution. *Proc. Biol. Sci.* **284**, (2017).

12. Yoshida, M.-A. *et al.* Gene recruitments and dismissals in the Argonaut genome provide insights into pelagic lifestyle adaptation and shell-like eggcase reacquisition. *Genome Biol. Evol.* **14**, evac140 (2022).
13. Taite, M. *et al.* Genome skimming elucidates the evolutionary history of Octopoda. *Mol. Phylogenet. Evol.* **182**, 107729 (2023).
14. Lindgren, A. R. & Anderson, F. E. Assessing the utility of transcriptome data for inferring phylogenetic relationships among coleoid cephalopods. *Mol. Phylogenet. Evol.* **118**, 330–342 (2018).
15. Anderson, F. E. & Lindgren, A. R. Phylogenomic analyses recover a clade of large-bodied decapodiform cephalopods. *Mol. Phylogenet. Evol.* **156**, 107038 (2021).
16. Lindgren, A. R., Pratt, A., Vecchione, M. & Anderson, F. E. Finding a home for the ram's horn squid: phylogenomic analyses support *Spirula spirula* (Cephalopoda: Decapodiformes) as a close relative of Oegopsida. *Org. Divers. Evol.* **23**, 91–101 (2023).
17. Chen, Z. *et al.* A genome-based phylogeny for Mollusca is concordant with fossils and morphology. *Science* **387**, 1001–1007 (2025).
18. Uribe, J. E. & Zardoya, R. Revisiting the phylogeny of Cephalopoda using complete mitochondrial genomes. *J. Molluscan Stud.* **83**, 133–144 (2017).
19. Strugnell, J. M., Hall, N. E., Vecchione, M., Fuchs, D. & Allcock, A. L. Whole mitochondrial genome of the Ram's Horn Squid shines light on the phylogenetic position of the monotypic order Spirulida (Haeckel, 1896). *Mol. Phylogenet. Evol.* **109**, 296–301 (2017).
20. Rosenthal, J. J. C. & Eisenberg, E. Extensive recoding of the neural proteome in

cephalopods by RNA editing. *Annu. Rev. Anim. Biosci.* **11**, 57–75 (2023).

21. Naef, A. (1923). Die Cephalopoden. Fauna e Flora de Golfo di Napoli. monograph 35, 1(1) part 2: 149–863

22. Carlini, D. B., Reece, K. S. & Graves, J. E. Actin gene family evolution and the phylogeny of coleoid cephalopods (Mollusca: Cephalopoda). *Mol. Biol. Evol.* **17**, 1353–1370 (2000).

23. Bolstad, K. S. R. Systematics of the Onychoteuthidae Gray, 1847 (Cephalopoda: Oegopsida). *Zootaxa* **2696**, 1 (2010).

24. Nixon, M. The Radulae Of Cephalopoda. *Smithsonian contributions to zoology* 39–53 (1998).

25. Kruta, I., Landman, N. H. & Tanabe, K. Ammonoid Radula. in *Topics in Geobiology* 485–505 (Springer Netherlands, Dordrecht, 2015).

26. Fuchs, D. Homology problems in cephalopod morphology: deceptive (dis)similarities between different types of 'caecum'. *Swiss J. Palaeontol.* **138**, 49–63 (2019).

27. d'Orbigny's (1841): d'Orbigny (1841). In: de Férussac, A. E. and d'Orbigny, A. (1835-1848). Histoire naturelle générale et particulière des Céphalopodes acétabulifères vivants et fossiles. pp. [1-96], i-lvi, 1-361, Atlas with 144 plates. Paris, Baillière.

28. Simakov, O. *et al.* Deeply conserved synteny and the evolution of metazoan chromosomes. *Sci Adv* **8**, eabi5884 (2022).

29. Ikegami, S., Takeda, Y., Mutterlose, J. & Iba, Y. Origin and radiation of squids revealed by digital fossil-mining. *Science* **388**, 1406–1409 (2025).

30. Fuchs, D. *et al. Longibelus* gen. nov., a new Cretaceous coleoid genus linking Belemnoidea and early Decabrachia. *Palaeontology* **56**, 1081–1106 (2013).

31. Schweigert, G. & Fuchs, D. First record of a true coleoid cephalopod from the Germanic

Triassic (Ladinian). *Neues Jahrb. Geol. Palaontol. Abh.* **266**, 19–30 (2012).

32. Košťák, M. *et al.* Rare Middle Triassic coleoids from the Alpine-Carpathian system: new records from Slovakia and their significance. *Swiss J. Palaeontol.* **143**, (2024).
33. Rieber, H. Cephalopoden Aus Der Grenzbitumenzone (Mittlere Trias) Des Monte San Giorgio (Kanton Tessin, Schweiz). Schweiz Palaeont. Abh. **93**, 1–96 (1973).
34. Suzuki, M., Iwashima, A., Kimura, M., Kogure, T. & Nagasawa, H. The molecular evolution of the pif family proteins in various species of mollusks. *Mar. Biotechnol. (NY)* **15**, 145–158 (2013).
35. Shimizu, K., Negishi, L., Kurumizaka, H. & Suzuki, M. Diversification of von Willebrand factor A and chitin-binding domains in Pif/BMSPs among mollusks. *J. Mol. Evol.* **92**, 415–431 (2024).
36. Zhao, R. *et al.* Dual gene repertoires for larval and adult shells reveal molecules essential for molluscan shell formation. *Mol. Biol. Evol.* **35**, 2751–2761 (2018).
37. Bai, Y. *et al.* Multi-omic insights into the formation and evolution of a novel shell microstructure in oysters. *BMC Biol.* **21**, 204 (2023).
38. Li, H. Protein-to-genome alignment with miniprot. *Bioinformatics* **39**, (2023).
39. Oudot, M. *et al.* The shell matrix and microstructure of the Ram's Horn squid: Molecular and structural characterization. *J. Struct. Biol.* **211**, 107507 (2020).
40. Mendes, L. F. S. & Costa-Filho, A. J. A gold revision of the Golgi Dynamics (GOLD) domain structure and associated cell functionalities. *FEBS Lett.* **596**, 973–990 (2022).
41. Sanchez, G. *et al.* The chromosomal genome sequence of the bigfin reef squid, *Sepioteuthis lessoniana* d'Orbigny, 1826 and its associated microbial metagenome sequences. *Wellcome Open Res.* **10**, 351 (2025).

42. Gavriouchkina, D. *et al.* A single-cell atlas of the bobtail squid visual and nervous system highlights molecular principles of convergent evolution. *Nat. Ecol. Evol.* 1–18 (2025).

43. Rogers, T. F. *et al.* Gene modelling and annotation for the Hawaiian bobtail squid, *Euprymna scolopes*. *Sci. Data* **11**, 40 (2024).

44. Destanović, D. *et al.* A chromosome-level reference genome for the common octopus, *Octopus vulgaris* (Cuvier, 1797). *G3 (Bethesda)* **13**, jkad220 (2023).
