# Supplementary material for: Rapid mid-Cretaceous diversification of squid and cuttlefish preceded radiation into coastal niches
Source: Nat Ecol Evol. 2026 Mar 30;10(4):662–76. doi: 10.1038/s41559-026-03009-1 (PMC13076209; doi:10.1038/s41559-026-03009-1)
Supplement: Supplementary file 2 — Reporting Summary [file 41559_2026_3009_MOESM2_ESM.pdf]

nature portfolio

Corresponding author(s): Dr. Gustavo Sanchez
Prof. Daniel Rokhsar

Last updated by author(s): Jan 26, 2026

Reporting Summary

Nature Portfolio wishes to improve the reproducibility of the work that we publish. This form provides structure for consistency and transparency in reporting. For further information on Nature Portfolio policies, see our Editorial Policies and the Editorial Policy Checklist.

Statistics

For all statistical analyses, confirm that the following items are present in the figure legend, table legend, main text, or Methods section.

n/a	Confirmed	
☐	☒	The exact sample size (*n*) for each experimental group/condition, given as a discrete number and unit of measurement
☒	☐	A statement on whether measurements were taken from distinct samples or whether the same sample was measured repeatedly
☐	☒	The statistical test(s) used AND whether they are one- or two-sided<br>*Only common tests should be described solely by name; describe more complex techniques in the Methods section.*
☒	☐	A description of all covariates tested
☐	☒	A description of any assumptions or corrections, such as tests of normality and adjustment for multiple comparisons
☐	☒	A full description of the statistical parameters including central tendency (e.g. means) or other basic estimates (e.g. regression coefficient) AND variation (e.g. standard deviation) or associated estimates of uncertainty (e.g. confidence intervals)
☐	☒	For null hypothesis testing, the test statistic (e.g. *F*, *t*, *r*) with confidence intervals, effect sizes, degrees of freedom and *P* value noted<br>*Give P values as exact values whenever suitable.*
☐	☒	For Bayesian analysis, information on the choice of priors and Markov chain Monte Carlo settings
☒	☐	For hierarchical and complex designs, identification of the appropriate level for tests and full reporting of outcomes
☐	☒	Estimates of effect sizes (e.g. Cohen's *d*, Pearson's *r*), indicating how they were calculated

Our web collection on statistics for biologists contains articles on many of the points above.

Software and code

Policy information about availability of computer code

Data collection	No Software was used
Data analysis	Genome annotation: Braker v3.06, Galba v.1.0.10<br>Transcriptome assembly: Mikado v2.3.4 pipeline<br>Annotation: InterProScan v5.71-102119, Diamond v2.1.8.162<br>Phylogenomics: Orthofinder v2.5.4, MAFFT v7.4.1, GBlocks v0.91b, Clan_Check v1.3, Phylobayes-MPI v.1.8, PAML 4.9, IQ-Tree v2.3.5<br>Gene Family: CAFE v5.1, PAML 4.9<br>Molecular dating: LSD2 v.2.4.4

For manuscripts utilizing custom algorithms or software that are central to the research but not yet described in published literature, software must be made available to editors and reviewers. We strongly encourage code deposition in a community repository (e.g. GitHub). See the Nature Portfolio guidelines for submitting code & software for further information.

Data

Policy information about availability of data

All manuscripts must include a data availability statement. This statement should provide the following information, where applicable:

- Accession codes, unique identifiers, or web links for publicly available datasets
- A description of any restrictions on data availability
- For clinical datasets or third party data, please ensure that the statement adheres to our policy

All the genomic and transcriptomic data generated from the newly sequenced species are found under the NCBI BioProject accession number PRJNA1332616.. Genomes generated by the Aquatic Symbiosis Project of the Wellcome Sanger Institute are available under the NCBI Umbrella BioProject PRJEB64979. The resulting alignments and phylogenetic trees are available at https://github.com/gushiro/decapodiforms_phylogenomics)

Research involving human participants, their data, or biological material

Policy information about studies with human participants or human data. See also policy information about sex, gender (identity/presentation), and sexual orientation and race, ethnicity and racism.

Reporting on sex and gender	N/A
Reporting on race, ethnicity, or other socially relevant groupings	N/A
Population characteristics	N/A
Recruitment	N/A
Ethics oversight	N/A

Note that full information on the approval of the study protocol must also be provided in the manuscript.

Field-specific reporting

Please select the one below that is the best fit for your research. If you are not sure, read the appropriate sections before making your selection.

☐ Life sciences ☐ Behavioural & social sciences ☒ Ecological, evolutionary & environmental sciences

For a reference copy of the document with all sections, see nature.com/documents/nr-reporting-summary-flat.pdf

Ecological, evolutionary & environmental sciences study design

All studies must disclose on these points even when the disclosure is negative.

Study description	We sequenced the genome of three squid species and performed a phylogenomic study that comprise a total of 16 coleoid cephalopods.
Research sample	The pygmy squid Idiosepius pygmaeus, the Japanese common squid Todarodes pacificus and the Ram's horn squid Spirula spirula were collected. Animal tissue were collected from anesthetized animals, and subsequently use for DNA/RNA sequencing.
Sampling strategy	Samples were obtained by catch or acquired in the super market.
Data collection	DNA/RNA sequencing was performed from the tissue collected.
Timing and spatial scale	I. pygmaeus was collected on November 2023, T. pacificus on October 2021, and S. spirula on February 2023. Mostly during the seasonal abundance.
Data exclusions	No data was excluded.
Reproducibility	Bootstrap support and Bayesian Markov chain Monte Carlo sampling
Randomization	N/A
Blinding	N/A

Did the study involve field work? ☒ Yes ☐ No

Field work, collection and transport

Field conditions	Samples were collected by catch during the collection of other animals. T. pacificus was collected from a vendor of a fish market.
Location	I. pygmaeus from Seragaki in shallow waters. S. spirula off Canary Island at the deep sea. T. pacificus from a fisherman.
Access & import/export	N/A
Disturbance	N/A

Reporting for specific materials, systems and methods

We require information from authors about some types of materials, experimental systems and methods used in many studies. Here, indicate whether each material, system or method listed is relevant to your study. If you are not sure if a list item applies to your research, read the appropriate section before selecting a response.

Materials & experimental systems

n/a	Involved in the study	
☒	☐	Antibodies
☒	☐	Eukaryotic cell lines
☒	☐	Palaeontology and archaeology
☐	☒	Animals and other organisms
☒	☐	Clinical data
☒	☐	Dual use research of concern
☒	☐	Plants

Methods

n/a	Involved in the study	
☒	☐	ChIP-seq
☒	☐	Flow cytometry
☒	☐	MRI-based neuroimaging

Animals and other research organisms

Policy information about studies involving animals; ARRIVE guidelines recommended for reporting animal research, and Sex and Gender in Research

Laboratory animals	N/A
Wild animals	I. pygmaus was collected with a net as by catch from shallow water. S. spirula was collected by catch using trawling. T. pacificus was collected from a tank of a fish market.
Reporting on sex	Morphological assessment of gonads. Animals were adults (age assessed by morphology).
Field-collected samples	Animals that were collected alive where maintained in oxygenated tank and anesthetized within 24 hrs.
Ethics oversight	Animals were anesthetized following ethical and welfare recommendations for cephalopod research.

Note that full information on the approval of the study protocol must also be provided in the manuscript.

Plants

Seed stocks	N/A
Novel plant genotypes	N/A
Authentication	N/A
